# Advancement of Sensor Integrated Organ-on-Chip Devices

**DOI:** 10.3390/s21041367

**Published:** 2021-02-15

**Authors:** Gabriel A. Clarke, Brenna X. Hartse, Amir Ehsan Niaraki Asli, Mehrnoosh Taghavimehr, Niloofar Hashemi, Mehran Abbasi Shirsavar, Reza Montazami, Nima Alimoradi, Vahid Nasirian, Lionel J. Ouedraogo, Nicole N. Hashemi

**Affiliations:** 1 Department of Mechanical Engineering, Iowa State University, Ames, IA 50011, USA; gaclarke@iastate.edu (G.A.C.); bxhartse@iastate.edu (B.X.H.); niaraki@iastate.edu (A.E.N.A.); taghavi@iastate.edu (M.T.); mehran@iastate.edu (M.A.S.); reza@iastate.edu (R.M.); nimaa@iastate.edu (N.A.); nasirian@iastate.edu (V.N.); lionelo@iastate.edu (L.J.O.); 2 Department of Materials Science and Engineering, Sharif University of Technology, Tehran 11365, Iran; niliparak@gmail.com; 3 Department of Biomedical Sciences, Iowa State University, Ames, IA 50011, USA

**Keywords:** organs-on-chips, biosensing, integrated sensors

## Abstract

Organ-on-chip devices have provided the pharmaceutical and tissue engineering worlds much hope since they arrived and began to grow in sophistication. However, limitations for their applicability were soon realized as they lacked real-time monitoring and sensing capabilities. The users of these devices relied solely on endpoint analysis for the results of their tests, which created a chasm in the understanding of life between the lab the natural world. However, this gap is being bridged with sensors that are integrated into organ-on-chip devices. This review goes in-depth on different sensing methods, giving examples for various research on mechanical, electrical resistance, and bead-based sensors, and the prospects of each. Furthermore, the review covers works conducted that use specific sensors for oxygen, and various metabolites to characterize cellular behavior and response in real-time. Together, the outline of these works gives a thorough analysis of the design methodology and sophistication of the current sensor integrated organ-on-chips.

## 1. Introduction

Organ-on-chip (OOC) devices first emerged into the scientific world around 1990 under the term “miniaturized total chemical analysis systems (µTAS)” [1], and have improved in sophistication, application, and popularity as time has progressed, with their potential being recognized. In the early stages of the development of these devices, they were limited in their application; however, as research and technology advance, so does the potential for these devices [2,3]. Recent efforts have allowed the potential of these devices to integrate sensors, allowing for real-time analysis of the biological processes—a breakthrough for the experimental world [4,5]. As an example of the progress, the Hashemi Lab worked on a sheathing fabrication technique and microfluidic device with optical sensors that could characterize micrometer-sized phytoplankton for gaining insight into global warming and ocean currents [6,7,8]. Microfluidic microfiber fabrication has made tremendous progress over the past few years, and can now serve as a scaffold for neural stem cells [9,10,11]. Microfluidic systems have started to be used in legitimate, helpful applications, such as in the creation of blood and urine assays, a paper-based microfluidic fuel-cell, and to study caffeine transport through the placenta during pregnancy [12,13,14]. Finally, as an affirmation of the promise of this technology, it is being used for early cancer detection by means of liquid biopsy research, and as an aid in disease diagnosis [15,16].

Among all of these applications, drug testing is perhaps the most important. The current drug testing regimen involves many trials and iterative steps to ensure they are safe, taking between two and ten years, and sometimes costing up to two billion dollars [17,18]. The current, and unfortunately well-vetted method for evaluating a new drug involves a fairly defined sequence of steps in which no steps should be omitted; this makes for a rather painstaking and process, and a low percentage of drugs that make it to being administered to a patient [19]. OOC devices with integrated sensors could potentially solve this long and expensive process by simply allowing for the characterization of a drug without many of the intermediate steps [5,20]. The OOC device resembles human organs or organ systems, so with proper analysis of the “organs” through sensor integration, the toxic effects could potentially be measured quickly and relatively inexpensively [21,22]. Within this ability to skip several time and money-consuming steps in the current process, concerns on animal testing, along with the care and expense to use live animals, could be eliminated [23,24]. 

OOCs can improve current drug testing methods, shifting away from animal testing, and provide more accurate reports of toxicological effects [25]. Another application of the OOCs with integrated sensors lies within the world of tissue engineering. Sensors, as will be outlined below, integrated into some OOCs, can characterize engineered tissue and tissue interactions with different stimulants in a way that improves the function and applicability of these substances [26]. OOCs can properly and promptly give insight into the interaction of the stress and strain of loading dynamics and drug-induced tissue degradation [5,27]. The nature of the OOCs also necessitate further advancement in tissue engineering as more physiologically relevant tissues are sought after to be used in these devices [5,28,29,30]. 

The methods and results of many current efforts that incorporate sensors within their OOC devices are outlined. Each study has been evaluated based on its advantages and disadvantages to better understand the current state of this rapidly advancing technology. Future opportunities and overcomings are used to conclude the current devices and methods discussed, portraying an updated vision of the potential of these OOC devices, such as in toxicological studies that can replace animal testing and for tissue engineering insight.

## 2. Sensor Systems

Research, such as that conducted by Zhang et al. and Bonk et al., has collectively given insight on a host of parameters via on-board measuring of pH and oxygen levels, in addition to other parameters, for proper cell characterization [31,32]. Both studies outlined in this section show how a system of sensors, and ideally a system of organ-on-chips (OOCs) also, is the future of the organ on chip technology.

**Figure 1:**
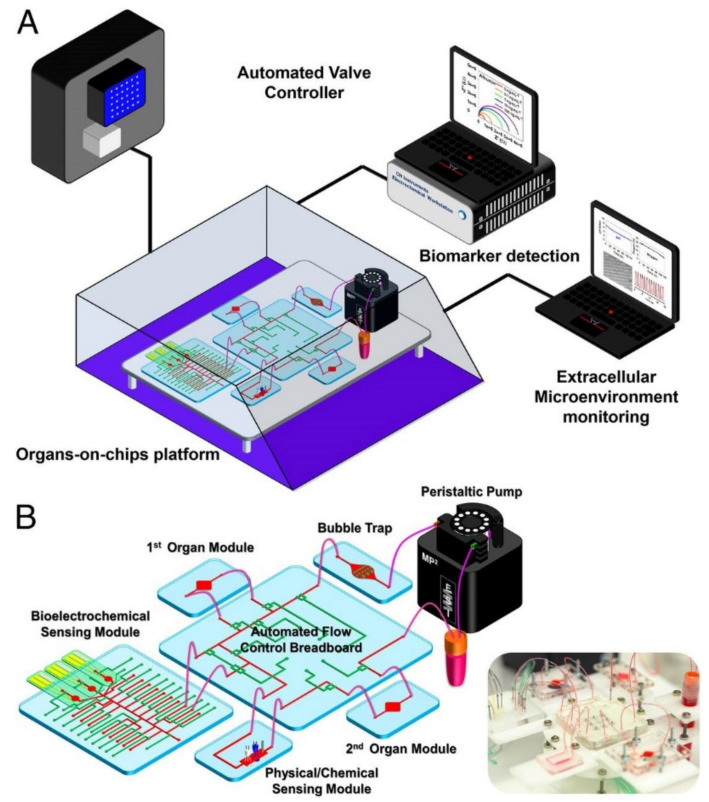
Multiorgan-on-a-chip platform with sensor systems, reproduced with permission from Ref. [31]. (**A**) The multiorgan-a-chip displaying the use of an incubator, pneumatic valve controlled that is automated, physical sensors operated by electronics, electrochemical signals measured by potentiostat, and a computer that analyzes commands. (**B**) The contents of the integrated microfluidic device, such as microbioreactors, reservoir, breadboard, bubble trap, electrochemical biosensors, and physical sensors.

Zhang et al. created an organ system-on-chip that is leading the integrated sensor on-chip technology through its sophisticated setup and capabilities. This platform effectively developed integrated organ-on-chip models with multisensory systems, allowing for further advances in drug screening and organ-on-chip model use [31]. Additionally, the system allows for complete automation after validation of the individual component functions, which is valuable in reducing human labor, especially with a high throughput or long-term analysis. The platform was capable of integrating OOCs units and modular sensing equipment was developed to promote the performance of current OOC devices for drug screening and real-time sensor applications through the in situ monitoring of biophysical and biochemical parameters. Within a benchtop incubator, including temperature and CO_2_ controls, the developed OOC platform was contained, and MATLAB code controlled the on-chip valving and the electrochemical station. A data acquisition card connected to LabView was used for physical sensing. Potential channel blockage and leakage were monitored by a flow sensor. The platform itself included a breadboard for the microfluidic routing, microbioreactors for organoid housing, physical sensors for measuring microenvironmental parameters, electrochemical sensing units for soluble biomarker detection, a medium reservoir, a bubble trap, and electrochemical sensing chips, which can be seen in Figure 1A,B [31]. The setup’s sensing capabilities were tested with automated drug screening of fully integrated human liver- and heart-on-chips. Long term monitoring for chronic drug responses were tested in the dual-organ human heart-and-liver-on-chips through acetaminophen (APAP) doses (0.5, and 10 mM) and the consequent detection of dose-dependent toxicity of the organoids. Similarly, the dual-organ human heart-and-liver-cancer-on-chips system was introduced to Doxorubicin (DOX), a chemotherapeutic drug (dose of 5 μM and 10 μM), to investigate the acute toxicity of the drug treatment.

The APAP doses introduced into the heart and the liver organoid system produced little disturbance, allowing for the continual physical parameter measurements of pH, O_2_, and temperature to be monitored stably over the entire time period. Additionally, dose-dependent toxic effects of APAP on hepatocyte survival were shown via end-point cell viability assessment. Furthermore, the electrochemical immunosensors achieved noninvasive biomarker analysis, revealing the levels of albumin and GST-α increased and decreased, respectively, depending on the dosage, with the higher concentration dosage causing a stronger effect; these results are consistent with the hepatotoxicity induced by APAP doses.

In the liver cancer group, human cardiac organoids (from iPSC-CMs) and human hepatic organoids (from HepG2/C3A hepatocellular carcinoma cells) were investigated for toxicity upon drug treatment while being linked together for 24 h [31]. Introducing DOX yielded significant death of the liver cancer organoid, as was expected. Lowered secretion of albumin and higher release of GST-α as also observed. Differing from the effects of APAP treatment, DOX administration induced cardiotoxicity, which was demonstrated by rounding, detachment, and death of the icardiPSC-CMs. CK-MB levels surged to high levels and were associated with the arrhythmic beating of the cardiac organoid.

The device proved to be versatile in its potential to be fit for different OOC devices, even with its drawbacks. Although the use of PDMS on the microfluidic chips has the potential to adsorb and absorb hydrophobic molecules and drugs, which was observed when higher doses of DOX were administered, the use of this material may limit the created platform function [31].

Another study that used pH and oxygen measurements in conjunction with other biological parameters was performed by Bonk et al. as a means of developing cell adhesion sensors [32]. They combined a multi-sensor glass-chip and a microfluidic channel grid to characterize cell behavior. The cells studied were mouse-embryonal/fetal calvaria fibroblasts (MC3T3-E1), and platinum electrodes were used to monitor oxygen (amperometric electrode), pH (potentiometric electrode), and cell adhesion (Interdigitated-electrodes structures (IDES)). On-chip electro-thermo micropumps were used to control flow in the microfluidic chip. The device allowed for testing of IDES with different spacing geometries’ (30- and 50 μm finger spacing) effect on MC3T3 cell proliferation detection sensitivity. In order to test for biocompatibility, microfluidics, and electrical sensing capabilities over time, the cells were cultured for 11 days in vitro. Various plots, such as capacitance measurements over time and current against potential were created and analyzed for the different IDES detection ability and for calibration of oxygen depletion in the media, respectively [32].

The oxygen sensor design represented that of a Clark-type amperometric sensor, but it lacked an oxygen-selective membrane. The sensitivity of the oxygen sensor was comparable to commercial systems. The pH sensor was characterized by potentials detected against an Ag/AgCl reference electrode between the pH values of 4 and 9. Measurements taken from both the IDES with different spacing suggests that increasing sensor area improves sensitivity, but it comes at the cost of oxygen attrition as high recording rates increase oxygen sapping. The individual IDES arrays allowed for the detection of cell distribution within the glass-chip system, and they could be cross-checked via microscope analysis [32].

The sensor and IDES developed proved successful in the detection of oxygen, pH, and cell proliferation, which are all important biological and physiological parameters necessary to understand for different testing applications. Although the data showed clear detection on the microfluidic device, many potential issues may limit the application. Primarily, the oxygen concentration for the device is limited; high cell densities were required to significantly reduce the oxygen level. This is a result of the high diffusion of oxygen through the system’s PDMS parts [33], and it prevents the correlation of respiration and acidification of the medium [32]. Conversely, the system provided clear advantages due to its sterile conditions in a simple incubator, and it led to clear improvements possible in future systems. The work also permits new experimental approaches for cell monitoring, e.g., co-culture of multiple cell lines and physical stimulation of cells, and ultimately helps pave the way toward replacing animal testing.

Bonk et al. created this apparatus and method in 2015, while Zhang et al. published their work, which recorded and characterized many of the same parameters, but with more defined intent and within a much more microfluidics sensor and organ system. The latter work seems to be setting the pace and making a profound impact on the sensor integrated OOC frontier, as is steadfastly showcased in many other current OOC-type reviews. These two studies show straightforward integration, and how multiparametric measurement may provide great insight into cellular activity. The studies to follow are presented similarly but fall within more specific categories, either a specific sensory type or for the purposes of sensing something specific.

## 3. Specific Sensors

### 3.1. Mechanism Specific Sensors

#### 3.1.1. Mechanical Sensors

Mechanical biosensors take advantage of more classical physics, such as stress and strain, through the physical alteration of material. These sensors aim to characterize material by providing insight into its mechanical properties [34]. The sensors present new integrated mechanical sensor technology that allows for the quantification and analysis of different biological membranes and the effect of drugs on certain cell types, namely heart, and liver. Additionally, further understanding of material behavior under different stresses and strains could lead to further innovation in tissue engineering, as it is seen to be a critical component in many biocompatible tissue engineering ventures, such as in bone reconstruction [35], heart valves [36], and cartilage [37].

Understanding the heart is a complicated task, but it is of the utmost importance as heart disease is the leading cause of death worldwide, and it has been for many years [38]. Heart disease is characterized by blood pressure, cholesterol, and artery blockage, which can cause myocardial infarction (heart attack) and even in some cases death [39,40]. The need for better drugs and understanding of heart disease prevention is apparent, and a heart-on-chip device of sorts that allows for monitoring of cardiomyocytes could help bridge the gap.

A recent study has developed a microphysiological system (MPS) that enabled the integration of soft strain gauge sensors that allow for the non-invasive readout of contractile stresses within the tissue [41]. This technology may allow for a new understanding of tissue morphologies, drug-induced functional and structure modeling, and pathogenesis, which could aid in the understanding and treatment options of heart disease, to name just one application.

Many MPSs currently fail to provide users with sensor integration, so this new facile fabrication process was developed for cardiac microphysiological devices through 3D printing. High conductance, piezo-resistance, and biocompatible soft materials were the basis for the design. Soft strain gauges were used within the biocompatible soft material, and micro structures were repeated to help guide the physio-mimetic laminar cardiac tissue self-assembly [41]. The sensors provided non-invasive, contractile readings of contractile stresses of tissue within an incubator. Direct ink writing multi-material 3D printing was used to create a device through the patterning of six different materials and the device was printed in a single continuous procedure. The inks developed were highly diluted to fit within the laminar cardiac tissue stress limits (1–15 kPa) [42,43]. Fabricated layers were between 0.5 and 6.5 μm thick, and the lateral dimensions were achieved by tuning the evaporation rate of the carrier solvent solution. A water-soluble, sacrificial release layer of the dextran was printed first, followed by the printing of cantilever bases (3 μm-thick), strain gauges (6.5 μm-thick), and wire covers (1.5 μm-thick) with thermoplastic polyurethane (TPU). Electrical leads were printed to the wires to integrate electrical measurement devices with the strain gauges [41]. The wire leads were covered with a printed insulating layer and 8 individually addressable wells were created. A grooved microstructure was printed to help the cardiomyocytes self-align as they would in native tissue [41], as cardiomyocyte alignment is functionally related to its contractability [44].

As done in previous works [45,46], using Stoney’s Equation, the resistance measurements were converted to strain measurements and a mathematical model was created to understand the readings [41]. Tissue twitch stress data were obtained and the results were similar to those of other studies [47,48], ranging between 7 and 15 kPa for laminar NRVM tissues. Optical tracking was also used to evaluate the stresses. To test the device, drug-dose studies were carried out with an L-type calcium channel blocker (verapamil) and the β-adrenergic agonist (isoproterenol). Laminar NVRM tissue was observed to have a negative chronotropic response to verapamil. Conversely, a positive chronotropic response for spontaneously beating laminar tissue was observed in response to isoproterenol. Both responses, to verapamil and the isoproterenol supported the reliability of the device as the results align with those of other sources [49,50]. The device also can test for changes in contractile stress of cardiac tissue over the course of several weeks. After 28 days, the longitudinal contractile stress increased (quickly between days 2 and 4), and then grew more steadily, while the spontaneous beating rate decreased. A significant increase in sarcomere length (1.7 to 1.8 μm) was observed between days 14 and 28.

This newer technology and setup is advantageous as it can support thicker micro-tissues; the MPS designed can work with the thickness of approximately 4 cell layers. The approach facilitates rapid customization, allowing for much more versatile use and a better future understanding of tissue engineering and drug screening.

To further improve understanding of engineered tissues sensor integrated organ-on-chip device (OOC) device was created to characterize membrane stiffness more fully. Membrane stiffness, whether for engineered body tissue or substrates used in device fabrication, is an extremely important physical characteristic as it affects many environmental factors and cellular interactions [51,52]. It also contributes to mechanical properties, such as in extracellular stiffness in morphogenesis [53], cell differentiation [54], and proliferation [55], as well as in the processes of tumor metastasis [56]. Membrane stiffness plays critical roles in many more biological applications, and as is seen from the few applications noted before, it is paramount in sustaining life.

The membrane stiffness of engineered tissues may now be better understood and characterized through the recent monitoring of engineered tissues with strain-sensor-laden microdevice arrays [57]. To do this, an OOC device was fabricated in which Hydrogels were covalently bonded to the bulging membrane of a developed OOC. In the deformable membranes, carbon dioxide nanotube (CNT)-based strain sensors were embedded to show strain-dependent electrical resistivity; the strain sensors were used to detect membrane stiffness through on-chip measurements of membrane deflection, where deflection was proportional to the sample tissue stiffness. Off-stoichiometry thiolone-based dimethyl siloxane (OSTE-PDMS) was carefully mixed and poured into an aluminum mold to create the bulging membrane platform with integrated sensors. After being molded, the OSTE-PDMS strips were bonded to glass substrates via spin coating and cured with UV light, and CNT:OSTE-PDMS blends (1:12 mixing ratio by weight) were used for strain sensors to be screen printed out of. The blends were cured over OSTE-PDMS membranes and then cured with UV light, with dimensions of 50 μm thick, 300 μm wide, and 1.4 cm long. Each deformable membrane contained one strip through the middle of it. Electrical connections were added and the membrane deflection magnitude and strain sensor signals are calibrated against applied pressure from actuation. MSCs derived from cryopreserved human bone marrow were seeded and cultured in the hydrogels; they were mechanically stimulated for 8 h/day for 15 days with 5% nominal tensile strain at 0.1 Hz. NEG-NB hydrogels were integrated into the OSTE-PDMS bulging membrane.

After device fabrication and setup, many tests were conducted, including on-chip tissue stiffness measurements, finite element analysis, mechanical compression testing, and confocal microscopy imaging. The strain and stiffness measurements are of particular interest because mechanical stimulation has been shown to affect cell differentiation [58], branching tube formation [59], and implanted tissue compatibility [60]. In the study by Liu et al., an increase in membrane deflection magnitude was observed from day 2 to 6 which indicated cell stiffness (9.3 ± 0.4 to 5.43 ± 1.02 kPa, respectively, and deflection magnitude increased after day 6). Conversely, the strain magnitude signal from the cell-seeded PEG-NB gels in static culture increased from day 1 to 15, corresponding to continuous gel softening [57], which is consistent with strain-softening expectations [61]. Static condition gels and stretched gels were softened at day 7, while cells in simulated gels were more spread at day 7; these trends were seen by the nuclear aspect ratio between static and simulated conditions (2.01 ± 0.05 to 2.19 ± 0.04), and a normalized average area of α-SMA increase further supported the findings. Cells that were stretch-simulated generated an increase in static culture to normalized areas of collagen (1.62 *±* 0.47 to 5.04 *±* 1.32 at days 1 and 7, respectively). The different gels gave way to different cell elongation. Expression of the α-SMA measured in culture was significant (3.25 *±* 0.11 to 7.01 *±* 0.58 to 8.54 *±* 0.41 on days 1, 7, and 15, respectively); stretching simulation had similar effects. Relative to the static culture, a 6-fold increase in collagen normalized area and integrated density was observed with stretching simulation. Related mechanically stimulated showed similar findings, with a positive correlation between a cell’s Young’s modulus and its spread area [62]. In situ monitoring of the compressive stiffness of MSC-laden PEG-NB gels under osteogenic conditions without simulation were monitored in the device array to prove its versatility.

The device allows for in situ continual measurement of hydrogel constructs. The deformable membrane platform with integrated strain sensors was developed that allows for 3D-hydrogel mechanical manipulation, such as compression or stretching while increasing the hydrogel construct stiffness in situ. The technique can be generalized to other OOC platforms to help with functional analysis and provide more insight into the dynamics of engineered tissue development.

Another study for membrane characterization was conducted by Jin et al. which allowed for monitoring of mechanically deformed cells and tissue and their effects specifically from hypertension [63]. Among other issues caused by cell and tissue deformation [64,65], hypertension is a common and serious problem as it may lead to ischemia, stroke, and poor cerebral circulation, diminishing cognitive ability [66,67].

The goal of this work was to integrate flexible and stretchable electrochemical sensors with good mechanical compliance into a microfluidic chip system for real-time monitoring of mechanically deformed cells and tissues [63]. The electrochemical sensor was constructed by conductive polymer-coated carbon nanotubes for mechanical compliance and electrochemical performance [32]. The circumferential stretch with different strains exerted on endothelium allied for simultaneous monitoring of stretch-induced signaling molecules that were released from the deformed endothelial cells. The sensor was prepared with a PDMS membrane between two microfluidic layers. Flexible electrodes were patterned at particular spots in the microfluidic device. A ribbon-shape flexible sensor was prepared and then bonded with two PDMS layers that each had microchannels. HUVECs were cultured on the electrode to form in vitro vascular endothelium. With a vacuum applied to the bottom channel, elastic deformation of the stretchable sensor was induced and biomechanical vasodilation was simulated, allowing the mechanically induces signals to then be monitored. Vasodilation, in this study, serves as an understood mechanical process, but it also has important implications in atherosclerosis and loss of blood flow in diabetic subjects [68,69].

The results show superior conductivity and mechanical tolerance for the electrode. The effects of mechanical deformations and electrochemical properties were tested by evaluating cyclic voltammogram (CV) shapes and current peak values when the electrode was under static or dynamic stretching with up to 100% strains; the results indicate that severe mechanical deformations have a negligible effect on the electrochemical properties. Through a 100 round fatigue cycle, the CVs were recorded and revealed that the device would withstand circumferential stress and retain its electrochemical properties. Reactive oxygen species (ROS) and nitric oxide (NO) were both chosen to be probe molecules for further characterization of the stretchable electrode, as they play roles in pathogenic processes and neurodegenerative disorders, respectively [63,70,71]. Amperometry results showed an observable 10.0 × 10^−9^ M response, with a 1.6 × 10^−9^ M detection limit calculated, thus indicating high performance of the electrochemical sensor with real-time monitoring of NO. ROS effects were investigated through monitoring the electrode’s response to H_2_O_2_ and amperometry results of 5 × 10^−6^ M H_2_O_2_ were observed with a detection limit calculated at 1.0 × 10^−6^ M. A thin polydopamine (pDA) layer was coated on the stretchable sensor which improved the hydrophilicity of the sensor. Through quantitative analysis, it was determined that 3–18% strain could be achieved by the device by regulating the airflow rate from 1 to 11 mL min^−1^ at a frequency of 0.1 Hz. 10% cyclic strain for 10 h caused cell alignment along the microchannel, which is what HUVECs behave like in real blood vessels. Statistical analysis showed that over 83% of the cells reoriented into an aligned state; this was visually confirmed with the staining of F-actin. Hypertension was reproduced by applying an 18% circumferential stretch on the HUVECs. L-NAME partially inhibited the amperometry responses, but a synergy of L-NAME and diphenyleneiodonium completely suppressed the amperometric responses, indicating that NADPH oxidase was activated to produce ROS through overstretching the endothelial cells. Overall, results indicate that the NO pathway could be triggered with a circumferential strain that exceeded a particular threshold, circumferential stretches at 10% strain could activate NOS to produce NO, and circumferential deformation of cells reaching 18% caused the simultaneous release of NO and ROS. ROS was determined to be more easily induced with circumferential strain, indicating the endothelial damage and dysfunction that occur under hypertension [63,72].

Circumferential stretch exerted on endothelium could easily be recapitulated by the system. The ROS and NO signals could be recorded from the induced stretch, which proves that the approach of this study could be used to gain insight into the biomechanical response to hypertension at a molecular level.

Current biological models have very low throughput due to the complexity and interconnected nature of biological processes in the body. Most models (such as cell culture models) are simple, single layer in vitro models [73]. There seems to be an exchange between accurate modeling and high throughput; the more accurate a model is, the less throughput it has potential for. Furthermore, Cook et al. described a five framework focus for creating and pursuing a successful project, and within their review, it was seen that a vast majority of toxicological studies failed [74]. OOC device technology is striving to overcome this obstacle through more accurate modeling of tissues, organs, or body systems, and the study described in the following may help close the gap between experimental and clinical drugs.

The study by Oleaga et al. was conducted over 28 days to investigate the cellular viability and function of a 4 human organ system consisting of liver, heart, skeletal muscle, and nervous system in serum-free conditions using a pumpless system [75]. A 28-day period was chosen because it is the minimum timeframe for animal studies to evaluate repeat dose toxicity [75,76]. By adapting bioMEM chips and a method of detection to work inside the microfluidic systems, it created a noninvasive technology platform that used a commercial amplifier to collect recordings. Each chip included ten electrons distributed into two rows. Primary hepatocytes, cryopreserved human induced pluripotent stem cell (iPSc) derived cardiomyocytes, cryopreserved human skeletal myoblasts, and expanded and differentiated cryopreserved human motoneurons were used for the liver, heart, skeletal muscle, and nervous system cells, respectively. The cells were cultured in the microfluidic devices inside an incubator for 28 days and were fed with a reduced osmolarity HSL3 medium, which was exchanged every 24 h through reservoirs. Using an inverted phase-contrast microscope with a 10× objective and AxioVision AC software, cell morphology was monitored and images of each chamber were taken daily to track changes. Hepatic markers, urea, and albumin were monitored from the daily medium exchange.

Twice a week starting on day four, after the assembly, cardiac, skeletal, and neuronal functions were measured, translating the amplitude of the CL displacement measured cardiac contractile activity from the induced contractile cells to contractile force. The electrodes also measured cardiac electrical activity that translated cardiac extracellular current differentials into field potentials. The multiorgan system was disassembled on day 28 followed by the cytochrome p450 enzymatic activity endpoint assays. This research furthers studies in chronic toxicity studies in vitro as an alternative to animal testing and for monitoring multiorgan function of long-term exposure to chemicals.

The results from the liver-on-a-chip experiments showed that throughout the four weeks hepatocytes maintained their specific morphology. Data were collected consisting of urea and albumin production. In the multiorgan system, albumin and urea production improved when compared to static monoculture controls. Additionally, the outcome of hepatocytes significantly improved when there was a presence of flow and other organ representatives in the platform [75,77]. The heart-on-a-chip experiments collected data on the electrical, spontaneous and stimulated, and contractile, mechanical, components of cardiac function. Data collection showed that throughout the experiment, beat frequency, conduction velocity, and QT-interval were stable for both spontaneous and stimulated activity. Although there was an increase in the mISI period, it did prove to be significant. Using Python, contractile activity was measured and showed after 28 days, in the multiorgan system, the cardiomyocytes contractile force was stable. The results for the skeletal muscle-on-a-chip experiments showed that since the myotubes were not innervated that skeletal muscle contractile activity stimulation was required. Under electrical stimulation, the contractile activity of the myotubes was observed and was stable throughout the length of the study. Data collected for the nervous system included electrical recordings, spike shape, amplitude, and rate parameters. In the neuronal chamber, shield barriers were incorporated which lengthened the cellular pattern lifespan, which later validated the system.

Maintenance of the neurons was demonstrated in the multiorgan system by representative images of neuronal morphology located at the top of the microelectrode array (MEA) electrodes. The results of the electrical recording showed only positive neuronal spikes with ≥10 µV amplitude and ≥0.1 Hz rate. Despite patterns over time that showed motoneurons were maintained, observations of morphological changes increased with longer and thicker axons. However, the measured functional parameters indicated the stable function of the neurons showed no significant impact. The technology has advanced as an alternative to animal testing models to perform chronic testing for human in vitro models, an important advance given the ethical and social concerns with animal testing [78].

The researchers successfully made modifications to reduce the size of the system, improve flow characteristics, and incorporate functional measurements for noninvasive monitoring of electrical and mechanical properties. Another advantage of their method was their sinusoidal rocking scheme, which allowed the system to better control the flow conditions within acceptable ranges. Although the common medium was not an optimal medium for each organ separately, it allowed function for all the organs in the multiorgan system. This system can provide a tool to model the PKPD profile of known drugs, and later predict the outcome of unknown drugs, similar to the results of other works [79]. Seeing as PKPD models are proving to be a promising technique for drug effects and efficacy, this integrated system shows particular promise [80,81,82].

Additionally, it has the potential to introduce studies such as comparing hepatic phenotypes, use iPSc derived hepatocytes, adding other organs in the coculture, drug interactions, chronic drug administrations, and personalized medicine. Currently, this technology helps us to perform studies that are required for the in vitro safety evaluations mandated by ICH guidelines.

From the mechanical sensors described above and their applications, it is easy to see that there is a place in the OOC sensor integrated technology for these sensors. While the previous studies were specific and immediately applicable, other studies have been conducted to specifically improve and test sensors that allow for the understanding of these important metrics. The study that follows, performed by Chen et al., highlights a new and applicable way to sense pressure, and thus unveil many biologically important processes and responses [83].

An on-chip pumping system consists of four solenoid-driven pumping units and four check valves were integrated onto a microfluidic circulatory system. The four created compartments resemble the four compartments in the heart, as the pumping system was designed to mimic the heart. The pumping unit was designed in a serpentine-like channel to allow for the pumping unit to serve all the culture a reason the device and the device was fabricated through the bonding of five PDMS layers in a 3D printed mold. Holes were punched out of the device to make check valve spaces. The multi-layer device was created in three steps: first, three lakers were bonded, with the first layer having been punched for control chamber inlets before bonding and the inlet and outlet punches were made for the fluid layer after bonding; second, layers four and five were bonded together, carefully allowing for holes of layer five to align with layer five pillars; Thirdly, the two layers from steps one and two were bonded together to complete chip. LabVIEW was used to control the solenoid valves operating through a pneumatic pressure controller; this allowed for precise pressure control within each chamber, a common technique of microfluidic control [84,85]. Cell culture medium was injected into the device, and once the fluid layer was completely filled, all ports were blocked to form a closed loop. A pressure sensor of flexible fused silica capillary was placed to measure pressure on the chip and then the pressure was then calculatable with various mathematical formulae. A PDMS chamber with a PDMS substrate device was created to evaluate the performance of the pressure sensor in terms of its sensing range, accuracy, reproducibility, response to pressure fluctuations, and long-term performance for cell culture applications; various tests were performed to evaluate these parameters. The microfluidic circulatory system was operated with physiological blood pressure conditions when the culture of human umbilical endothelial cells (HUVECs) were on the chip. Images were taken every 3 h with an inverted microscope while the boundaries of the cells were outlined with ImageJ (NIH). Cells were stained and imaged with fluorescence microscopy for visual assessment.

The sensing range was determined through the application of a stepwise function from 0 to 142.5 mmHg. The liquid–gas interface movement was recorded and processed to gather the measured pressure. Pressure variation within 5% was determined from the measured and applied pressure values over the entire range. The sensor detected a maximum pressure of 134.5 ± 0.3 mmHg and a minimum pressure of 1.5 ± 0.2 mmHg; doth measured with very little error, showing the system’s ability to monitor abrupt pressure changes. The data of the pressure recordings showed the sensor could measure pressure at least 1000 times without variation of more than 5%. Furthermore, the sensor was tested for 7 days with a culture medium at high humidity and 37 °C with small variations tested, showing the sensor has long-term reliability. Through precise chamber control, the user of the sensor can adjust the pressure and heartbeat rate by changing the applied pressure and solenoid opening valve frequency. Mimicked left ventricular pressure (LVP) peaked at approximately 123 mmHg, end-diastolic LVP was approximately 9 mmHg, and arterial pressure was mimicked at 115 mmHg/69 mmHg (systolic/diastolic) at 75 beats per minute (bpm), which was consistent with other control group studies [86,87,88]. The HUVECs were cultured inside the cell and analyzed for cell elongation, with the shape index (SI) used to quantify the analysis, showing that the cells were slightly elongated without shearing. Conversely, epithelial cells became significantly elongated at 8 h compared to 0 h (SI of 0.49 to 0.59). Further analysis showed that cells became more elongated over time, while the angle of orientation of the cells did not show a significant change over the 8-h testing period.

The sensor features “plug-and-measure” capabilities, in which a user can choose the point of interest within the PDMS microsystem by simply inserting the capillary with the aid of a needle; thus, allowing great facilitation into microsystems. The device allows, through the control of the pressure environment in vitro, the ability to study biological and cell behavior processes, and more specifically, the device could be used to mimic cardiac dysfunction conditions, such as hypotension. The on-chip pumping system enables the circulatory system to be a close replicate of in vivo environments, providing more meaningful results when compared to other steady-flow devices.

#### 3.1.2. Impedance Sensors

Impedance based biosensors are used in many different realms of the scientific and sensing worlds, such as substance toxicity [89], virus detection [90], and skin hydration monitoring [91], to name a few. Resistance measurements can characterize many different biological structures and their roles and have been particularly useful in characterizing cell membrane thickness and cytotoxicity, which can be seen in many of the studies analyzed in the following. Within microfluidic biosensing, Transepithelial Electrical Resistance (TEER) impedance-based biosensing is particularly useful as It is used to measure tight junction dynamics of epithelial single-layer models [92], and they are trusted for reliability and accuracy [93]. The capacity of this measurement technique allows for further understanding of drugs, membrane wear, and exposure effects on epithelial layers. Many of the current organ-on-chips (OOCs) are integrated with this biosensing technology, providing the potential for insight into cell barrier function.

**Figure 2:**
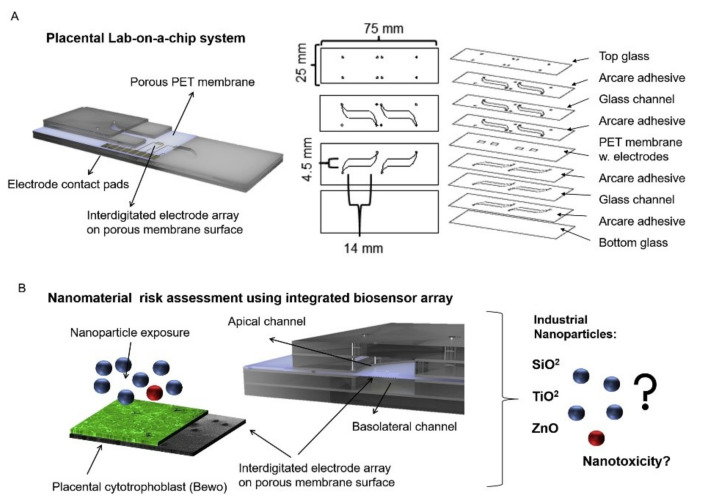
A lab-on-a-chip experiment investigating how nanomaterial can negatively impact a placenta, reproduced with permission from Ref. [94]. (**A**) Fabricated porous PET membranes are located under an impedance biosensor array. (**B**) To test the lab-on-a-chip, a nanomaterial risk assessment at the placental barrier is conducted.

Nanoparticles can be absorbed into the body through digestion, inhalation, or dermal uptake, which can serve detrimental effects on people’s health [94,95]. This research specifically focused on the transportation of nanoparticles through a placenta, which can have negative effects such as placental and fetal inflammation, fetal growth restriction (FGR syndrome), and fatal infections [94]. The negative effects of nanoparticles into the placenta are not foreign as they are seen by well-known detriments of alcohol [96], to the less well-understood effects of caffeine [12]. It is important to understand the effects of these substances as it affects fetal health. Schuller et al. developed a placenta-on-a-chip, containing an array of interdigitated impedance biosensors to investigate nanomaterial risk assessment studies for placental cell barriers, which can be seen in Figure 2A,B. Nanoparticles that were used in this study were silicon dioxide (SiO_2_), titanium dioxide (TiO_2_), and zinc oxide (ZnO), which are encountered frequently in everyday life, such as in sunscreen and many foods (colors, flavors, and preservatives) [97,98]. Observations were made from fluorescent dextran permeability assays and tetrapolar trans-epithelial resistance measurements [94,99]. Schuller et al. used BeWo trophoblast cells and data were collected based on the ability of the cells to attach, spread, and proliferate on the membrane surface [94]. Immunofluorescence staining was performed and showed that in the presence of the cell line a merged layer occurred, which agrees with patterns in unmodified porous membranes. To ensure that their membrane-bound electrode method worked for molecular transport research, apical-to-basolateral molecules were put through the in vitro placental epithelial barrier cell. Through observations, they found that after 2.5 h of exposure to 0.11 ± 0.04 μg/mL of sodium fluorescein allowed smaller molecules to more easily cross through the placental barrier than 0.04 ± 0.01 μg/mL 20 kDa and 500 kDa Dextran. These data indicated that their procedure had no significant impact on cell–material or biomolecule–cell interactions at the membrane biointerface. To detect cell barrier function, additional electrodes were integrated into their membrane-bound electrodes located to the top and bottom of the glass slides. Those results showed that after 24 h of exposure to 21 nm hydrophobic Al-coated titanium dioxide, and 20 nm amorphous silicon dioxide nanoparticles there was no loss in barrier integrity which could be confirmed by metabolic Presto blue viability assay. After four hours of exposure, there were near levels of reactive oxygen species in both nanoparticle types; however, when exposed to 80 nm zinc oxide nanoparticles there was a significant acute cytotoxic after four and 24 h of exposure. After four hours of exposure to 3mM zinc oxide nanoparticles, there was a decline of 99 ± 0.15 % of normalized impedance, and after 24 h of exposure to 770 μM and 250 μM ZnO, normalized impedance declined by 0.97 ± 0.08 % and 66 ± 0.19 %. These results were again similar to those from PrestoBlue viability and Reactive Oxygen Species (ROS) image data.

Since the membrane could be altered, researchers tested the physical, chemical, and biological properties of their integrated porous membrane to ensure accurate results. They found that at the membrane biointerface cell–material and biomolecule–cell interactions were not significantly influenced by their metallization procedure or electrode fabrication procedure. Their study is unique in that they integrated sensors on top of the porous membrane, which separates circulation systems between the mother and fetus. However, limitations to their study include the use of TEER measurements, which include the lack of spatial resolution, parallelization, and assay reproducibility. In addition, due to manual electrode handling, it can cause experimental inconsistencies. Despite these limitations, TEER is becoming popular to use in cell barrier analysis. Integration of electrodes in microfluidic devices also has some limitations, such as connectivity, device geometry, and poor time-resolved read-out. However, the results of their studies followed traditional TEER readings and are consistent within vitro/vivo reports, PrestoBlue viability, and ROS image data, which show promising applicability, and specifically shed light onto nanoparticles interaction with placental trophoblasts.

An effort to understand the specifics of barrier function began by Van der Helm et al. creating an OOC device to help monitor cell layer characteristics. The OOC was developed by placing a PDMS membrane between a PDMS top channel and PDMS bottom layer and then adding a PC with electrode layer on each side to create both a four and six electrode layer [100]. The cells (human adenocarcinoma cell line Caco-2) were cultured on the membrane as either a flat monolayer when cultured statically or in villus structure when cultured under flow [100]. Impedance spectroscopy measurements were taken using an Autolab PGStat12 machine over 12 days. These were taken immediately after the OOCs were taken out of the incubator (~2 min) to limit the effect of pH changes from the pCO2 in the ambient air. Two different current density data and the input current were recorded and allowed for the sensitivity distribution of the cell layer to be calculated. The sensitivity distribution was then normalized with the cell layer elements and the resulting distribution provided insight into how each part of the cell layer contributes to the total measured resistance. Impedance measurements were also taken to check for convergence; MATLAB R2016b was used for processing the model of that data. Galvanostatic impedance spectroscopy was mimicked to analyze the cell layer resistance and capacitance.

The impedance spectroscopy measurements combined with electrical simulation made it possible to carry out both TEER measurements and to assess the differentiation state of human intestinal epithelium within the OOC device. Previously, TEER measurements have been limited in application because they are influenced by non-biological parameters [100,101]. However, with the combination of electrical stimulation with input TEERs (0 Ω cm^2^ to 1000 Ω cm^2^), a developing culture cell layer was modeled. Epithelial resistance values were plotted against the input TEER, creating a calibration curve that showed the effective area associated with cell layer resistance and allowed for TEER normalization. Measuring capacitance and villi area ratios over the 12-day culture period showed that increased capacitance appears to be a direct result of villi formation. The cell measurements were obtained to capture the capacitance of the lipid bilayer membrane that separates the extracellular and intracellular conducting media. Both the resistance and capacitance from the cell layer were derived directly from the spectra measurements. Due to different resistive behaviors at different frequencies, the contributions of the cell layer to the total measured resistance were distinguishable.

The methods used in the study were the first done in a manner that exploited the frequency domain data to assess differentiation and tissue barrier function. Unlike conventional single-frequency TEER measurements, the methods used to eliminate the need for measuring blank chips as control measurements. Furthermore, the methods may be adapted for any OOC device to quantify changes in tissue differentiation and changes in tissue barrier function in real-time. The electrical stimulation enabled the measured cell layer resistance to Teer values to be normalized, thus allowing for comparison between other normalized measurements on different OOC devices.

Another study that was conducted in an effort to understand the human heart was conducted, but this time the researchers studied the combination of TEER and multi-electrode array (TEER–MEA) on a chip. This was done by Moaz et al. to further knowledge in drug effects and biological processes of the heart, specifically with human cardiomyocytes [102]. The chip was composed of a porous (PET) membrane that separates two parallel hollow barrmyocardium by exposing the chip to pro-inflammatory tumor necrosis factor-alpha (TNF-α) and isoproterenol, which are connected to cardiac disease and treatment of bradycardia. Data from these assessments included measurements of electrical activity, barrier function, and conformational changes of the cell monolayers.

Pt black electroplating treatment was used by the researchers to verify their system instead of using untreated platinum electrodes due to its ability to stay within the required electrophysiological parameters. Testing of the TEER values showed that there was a steady increase and stabilization: the values averaged 232 ± 47 Ω on day two and at the end of the six days the endothelium showed stability over the entire experiment. Before seeding, the culture membrane was 1190 ± 194.63 nF and declined to 226 ± 89 nF after seeding, which occurred on day two. This pattern of decreased levels continued for the remainder of the testing. A significant disruption of the endothelial batter was observed when TNF-α (2 μg mL^−1^) was administered and TEER values declined after 24 h from 230 ± 45 Ω to 15 ± 13 Ω. However, there was an increase in capacitance from 194 ± 33 nF to 2968 ± 52 nF, which correlates with other reports. Data of microelectrode array (MEA) measurements resulted in a 60 beats per minute average basal beat rate, and a 420 ± 5 ms basal rate-corrected field potential duration, which is slightly lower than the average heart rate found in one study [103]. These values for the beat rate and field potential duration are similar to those previously reported [102]. With MEA measurements, conduction velocity was calculated to be 66.6 ± 4.5 μm ms^−1^, which again agrees with previous reports.

The beat rate increased by 80% while the corrected field potential duration (cFPD) rose by 90% when isoproterenol was administered into the basal myocardial channel. However, there was no significant change in beat rate or cFPD when it was administered into the endothelial channel. TEER values were also measured in addition to the changes monitored in the cardiac layer using MEAs to investigate if isoproterenol alters the endothelial barrier. The results showed that although isoproterenol heavily influenced the cardiomyocytes, it did not impact the endothelial barrier, which was expected by the researchers, assumedly based on the previous findings of isoproterenol [104].

This study showed that, through the use of the created dual sensor system, there is an ability to monitor both endothelial barrier function and electrical activity of the cardiomyocytes simultaneously. Additionally, they added a step in the multi-electrode fabrication by integrating a signal enhancing electroplate. However, since pyknotic nuclei in the monolayer were not observed, their results indicated that TNF-α dose did not result in endothelial apoptosis, contrasting commonly known effects of TNF-α [105]. This real-time dual-analysis may allow for a better understanding of the exact alterations on membrane function caused by certain stimulants, as in the case with TNF-α and isoproterenol in this study.

Electrically active cells were studied in this next study by Henry et al. A 4-electrode chip capable of measuring TEER and cell layer capacitance was developed and integrated into a microfluidic device that contained upper and lower channels separated by a membrane (PET membrane for human lung small airway chip or PDMS membrane for human Gut Chip) [106]. A polycarbonate substrate served as the base for the electrodes (1 mm wide with 1 mm spacing) to be positioned on. The electrodes were fabricated and positioned in a way that made them physically stable, and they were transparent, which allowed for qualitative optical microscopy assessment of the cells. TEER measurements were taken to assess barriers both in the mucociliary epithelium and in the human Gut Chip lined with intestinal epithelial cells. To test the legitimacy of the TEER results as measurements of tight junction cell layers (impedance), EGTA, a Ca^2+^ chelator, was introduced after day 6 and a clear drop in impedance was measured; impedance measurement values rose when the EGTA was removed, thus proving the TEER measurements were legitimate, as they aligned with expected and tested reactions to the EGTA [107].

TEER measurements were recorded on days 1, 4, 6, 17, 22, 46, and 62. Resistance and capacitance values were relatively constant between days 1 and 6, at 200–600 Ω and 0.5 n, respectively. For resistance measurements, an air-liquid interface (ALI) was created on day 6 and the resistance measurements consistently rose to around 1700 Ω on day 62, indicating epithelial growth. After day 5, the capacitance measurements were recorded as greater than 0.9, indicating the cells formed a mature monolayer with behavior like that of an ideal capacitor. It is important to note that unlike other studies conducted that yielded Ω *cm^2^, the results of this study report resistance purely in Ω. The other recordings in other studies were conducted to normalize the resistance data, but the results may be skewed by the hand-manipulated electrodes, so the normalized results have somewhat unclear implications. Additionally, the area component of the normalized measurement indicated homogeneity across the cell, and that was not assumed in this study. The pure resistance measurements do prove advantageous in this study because they were consistent between the measurements of the two tests performed, therefore indicating the chip design and electrode placement is robust.

The TEER measuring chip technology designed and discussed may lead to new organ-on-chip applications, such as measuring short circuit currents or action potentials of electrically active cells. A test on an electrical double layer epithelium confirmed the generalized idea that the TEER chip design could apply to other cell types and across other microphysiological systems. Finally, the polycarbonate fabrication chosen for the design has clear advantages over glass, such as its easy pattern ability and subsequent low cost, and it does not need additional boundary layers. This electrical activity is an effective way for cells to communicate; action potentials are used to transmit signals [108], and in that sense it is similar to the following study.

The research was conducted on spheroids to collect data of tissues through measuring electrical activity in hopes of better understanding the cell–cell communication process that would be useful for research in treating diseases, such as arrhythmias [109]. This activity was measured on 12 channels of 3D self-rolled biosensor array (3D-SR-BA) surrounding spheroids. 3D-SR-BA are carefully rolled to create curvature, which improves biosensor-cell interface, and are fabricated using specific microfabrication techniques. Biocompatibility analysis was then performed using live/dead assay kits to measure cell viability, a common technique for both qualitative and quantitative analysis [110]. To improve recordings, MEAs were fabricated on 3D-SR-BAs to study the cellular network and were modified as well with a porous conductive polymer, poly(3,4-ethylenedioxythiophene): poly(sodium 4-styrenesulfonate) (PEDOT:PSS). It was modified with PEDOT:PSS because it was able to work as a transistor channel and as a support for cell attachment in 3D. The electrical and optical analysis was performed using the Intan acquisition system, which found field potentials (FP), and time latencies. These data were then later analyzed using a custom MATLAB script and a statistical analysis t-test was used to assess if there was significance with the modifications of PEDOT:PSS deposition, and encapsulation of 3D-SR-BA.

When comparing non-capsulated spheroids and capsulated spheroids there was no significant difference in percent viability, meaning 3D-SR-BA does not impact cell viability. When the research was performed individual ionic currents of Na+, K+, and Ca^2+^ across the cell membrane were examined. Those specific currents were used because Na+ showed upstroke, K+ showed repolarization, and Ca^2+^ showed plateau phase/beat frequency in the electrical recordings which indicated field potentials (FP) and time latency [109]. Consistent with other publications, channel four had stable recordings with an average FP amplitude of 1314 ± 25 5 µV and FP duration (FPD) of 279 ± 29 ms (*n* = 100 peaks). When the spheroids were encapsulated with 3D-SR-BA for three hours, the recordings were also stable. Time latencies were clear between FP’s with an average of 12.45 ± 1.88 cm/s, 6.09 ± 0.65 cm/s and 14.55 ± 1.79 cm/s. Although the averages were different, each spheroid had consistent time latencies throughout each recording and provided high spatial/temporal resolution in 3D.

3D spheroids may be more accurately studied than 2D platformed cell cultures because they resemble cell-to-cell communication more effectively. The material surrounding the spheroids, 3D-SR-BA, is biocompatible, does not impact the functionality of a spheroid/viability of cells, can be studied for several hours, and produces high spatial and temporal resolution in 3D, which cannot be achieved using Ca^2+^ transient imaging, thus showing the immediate application and improvements made by using this impedance-based biosensor within an OOC device.

As mentioned earlier, toxicological understanding is of paramount importance in the current medical world, and impedance-based biosensors prove to be another effective way to quantify the effects of toxic exposure on epithelial layers, as will be seen in the studies outlined below. The technology for impedance-based sensing has previously been proven effective [99,111], and the following studies have successfully integrated their OOC devices to further prove their effectiveness and new versatility within the microfluidic devices.

To reduce the role of animal testing and to gain more accurate drug/chemical toxicity data relating to the liver, researchers conducted a study of a liver-on-a-chip device incorporating physiological sensors [112]. Their device is a microfluidic 2D monolayer cell culture system, which was made from a glass chip consisting of top and bottom ITO-based embedded electrodes to measure TEER values. Those values were monitored using software based on LabVIEW. Photoelectric pH sensors were also connected to the device to measure electrical impedance and pH. Over three days, researchers tested toxicity by administering different concentrations of doxorubicin, epirubicin, and lapatinib. Data on TEER values, pH, and other biological markers were collected, for the researchers to study the acute metabolic and physical response of cells. Colorimetric, enzyme-linked immunosorbent assay (ELISA), confocal assays, and cell viability assays were also performed, further showing the liver-on-chip device provides real-time online data on drug-induced liver injury in vitro.

After 24 h of exposure to the drugs, data were collected from the sensors, specifically, the pH values were collected every hour. The results showed a significant decreasing trend in the slope of the non-linear data curve when exposed to higher concentrations of the drugs. When compared to the control group, without drug treatment, the pH data had a higher decreasing slope. These data indicated that varying concentrations of the drug impacted cell growth, resulting in acidification of the cell culture media. Higher cell death rates were encountered when higher concentrations of the drugs were administered. TEER values were collected similarly and impedance frequency was recorded at a fixed frequency of 60 Hz indicating that as time increases, impedance increases due to the cellular growth and the formation of cell–cell tight junctions. A data plot was made to reflect the impedance versus frequency (log10) and showed a characteristic exponential pattern. In the control group, TEER values showed a linear pattern and lower electrical impedance. Cellular impedance magnitudes were further utilized for computing relative CI.

The results of CI and Live/Dead cell assay were compared, and the researchers claim that their method can be used to indicate acute cytotoxicity for new drugs. In addition, microscopic images were taken at different time intervals to examine the cellular growth resulting in finding that there was a significant correlation between TEER impedance and cell growth. Further tests were conducted, which observed albumin and lactate concentrations. Data of the concentrations demonstrate the physiological activity of the liver on-chip device. In conclusion, the researchers found that higher drug concentrations resulted in higher cytotoxicity of their liver-on-chip device, and pH decreases and as time increases.

The researchers chose to collect data regarding albumin production because it is considered to be a primary biological marker of the liver and liver cell physiology is directly proportional to the rate of synthesis. Results also demonstrated complete cell monolayer formation, metabolic activity, and high TEER values, which confirms the accuracy of their liver-on-a-chip device. In addition, the microscopic images compared to biochemical assays further confirmed the quality. It should be noted that low TEER values reflect the poor quality of cultured cells. Their results are collected through a controlled environment of the glass microchips, which is an advantage because temperature variation was found to significantly affect TEER measurements.

These findings and methods are similar in scope to the work conducted by Khalid et al., as they conducted testing on an OOC platform, specifically a human lung, integrated with TEER impedance and a pH sensor to observe the cytotoxic activity of drugs [113]. In addition, a 3D-printed digital microscope was used to visually monitor the cells on a chip, which has not been done before. This study utilized lung cancer NCI-H1437 cells in their study to test varying concentrations of doxorubicin (DOX) and docetaxel, which was measured by the TEER impedance sensor. The cell index (CI) was calculated from the recorded data and the optical sensor was used to collect data regarding the media pH. Data collected during the drug treatment included pH response and microscopic images. The researchers then conducted a live/dead assay at the end of the experiments to compare cell viability.

A series of tests were conducted over a time period of 2 days on four similar lung-on-a-chip devices equipped with the TEER impedance sensor and pH sensor. Each drug, doxorubicin (DOX), and docetaxel was tested three different times with varying concentrations, and data were recorded for 24 h after each administration. DOX used concentrations of 5, 7.5 and 10 μM, and docetaxel used concentrations of 0.1, 0.3, and 0.5 nM concentrations. Data were collected every 30 min for the pH sensor, and the TEER sensor was set to stay at a constant 60 Hz. To observe any frequent changes, on hour 6, 24, and 36, microscopic images were taken.

The TEER impedance data indicated that an increase in the concentrations of the drug resulted in higher cell death rates, specifically DOX in a comparison between docetaxel, based on CI measurements. CI measurements were later calculated at the end of the experiment for this particular sensor and a graph was constructed showing the relationship between impedance and frequency. The range of the pH was 6.0-8.5 and sensitivity was found to be 489 ± 17 mV/pH, which is much higher than a previous report [113]. Results of the pH showed that it was observed that as concentrations increased for DOX, pH decreased: (−0.034, −0.036, and −0.044 pH/h for 5, 7.5, and 10 μM DOX, respectively). Docetaxel yielded different treatment results. The researchers hypothesized that DOX had higher rates of decreasing pH because there is a higher release of acidic species upon cell death. During the administration of the drug treatment, microscopic pictures were taken, which show cell growth and death. It was noted that density decreases slowly in some areas as time increases; thus, showing the possible detachment of dead cells. After 54 h, the control chip had an 81% increase in CI, while the other chips showed a decrease in CI value regarding increases of DOX: 57.3% at 5 μM, 47.4% at 7.5 μM, and 35.5% at 10 μM. Similar results were found for docetaxel: 37.74% at 0.1 nM, 31.24% at 0.3 nM, and 22.6% at 0.5 nM. When compared to the live/dead assay, the CI value reflected a lower cell rate of death. Despite this, TEER impedance data and live/dead assay had similar trends regarding cell viability due to the presence of varying concentrations of DOX and docetaxel.

The limitations of this study include that the CI values and live/dead assay were different, which could be due to the drawbacks to live/dead assays. In addition, the trial of 10μM DOX had a notable high CI value when compared to the live/dead assay; however, this could be the result of dead cells being attached to the collagen. Despite the drawbacks, this research is applicable and relevant in studying the future microphysiological systems and development of personalized medicine due to its evaluation of cytotoxic in novel drug compounds, hopefully increasing drug testing and viability on the market earlier on.

The final impedance-based biosensor analyzed is one that uses TEER measurements within a multi-tissue OOC device for understanding system toxicity. This kind of system leads the OOC technology as it allows not only the understanding of one organ type but for related (or seemingly unrelated) systems. Zhang et al. designed a system with similar implications [31], as described above. In this work by Skardal et al., a three-tissue organ-on-a-chip system was created, consisting of liver, heart, and lung, to observe the inter-organ interaction when drugs are administered, which may further bring understanding on interrelated effects of drugs on these organs [114,115].

Each organ is represented using a tissue model from human tissue organoids [116]. The liver specifically used primary human hepatocytes, stellate cells, and Kupffer cells, and the lung tissue model incorporated TEER electrodes. Each spheroid organoids was bioprinted using fluidic device technologies to integrate the tissue organoids. Testing consisted of toxicity analysis in the liver-on-a-chip and heart-on-a-chip, inter-organ interaction of liver and heart-on-a-chip, the function of lung-on-a-chip, and inter-organ interaction of liver, heart, and lung-on-a-chip.

The liver-on-a-chip model was first developed, and toxicity was measured when exposed to acetaminophen (APAP) and the clinically used APAP countermeasure, *N*-acetyl-L-cysteine (NAC). Testing consisted of 1 mM APAP, 10 mM APAP, and 10 mM APAP +20 mM NAC for a 14-day experiment. The control group had a high level of viability of 70–90% and the 1 mM APAP group showed the viability of 30–50%. The 10 mM APAP group had little to no viability of 0–1%, while the 10 mM APAP +20 mM NAC had a viability of 50–60%.

After viability assessment, researchers conducted albumin analysis, which showed a constant albumin production until drug administration. On day 6, albumin levels significantly declined in the 10 mM APAP group compared to the 0 mM control and 1mM APAP group; at the end of the experiment, the albumin levels were barely measurable in the 10 mM APAP group. On the other hand, levels in the APAP + NAC organoid were significantly greater and nearly similar to the control levels. The general pattern of these tests shows that there is a significant cytotoxic response to APAP and NAC treatment. Different concentrations of epinephrine (0, 0.1, 0.5, 5, and 50 µM) were tested to see the lowest concentration needed to increase beating rate in the heart organoid, with results showed beating rate increased with an increase in the dose but reached a plateau at 5 µM. Next, different propranolol concentrations (0, 0.5, 5, and 20 µM) were induced into the cardiac organoids and after an incubated time period, epinephrine was added at 5 µM, with results showing that increasing concentrations of propranolol more effectively prevented epinephrine-induced increases in beating rates. Next, the researchers studied inter-organ interactions with a liver and heart-on-a-chip where a cardiac construct was dependent on the metabolic capabilities of an upstream liver construct. In this model, in-line sensors were combined with the microfluidic components in the form of an electrochemical sensor component with the intent to provide constant measurements of up to three soluble biomarkers at a time. The same tests of epinephrine and propranolol were used and first tested independently in a cardiac-only system and then used on the dual organoid system. Liquid chromatography-mass spectrometry (LC-MS) was performed using media aliquots confirmed that the liver metabolized the propranolol. A lung-on-a-chip was developed and successfully was able to monitor organoid integrity and changes in function. Finally, researchers developed a three-tissue liver, heart, and lung organ-on-a-chip that studied the administration of bleomycin. On day 3, bleomycin was infused for a total of 6 days. Relatively low numbers of dead cells were found in both the no drug control groups and the bleomycin-treated groups when researchers conducted LIVE/DEAD analysis. However, cardiac organoids appear less tightly aggregated when exposed to bleomycin, so researchers conducted another test on just the cardiac-only system. The results showed bleomycin did not cause cessation of cardiac organoid beating, indicating that bleomycin may induce production of a secondary factor from one of the other tissues in the platform, which would later be found to be lung organoid. Other experiments were performed such as administration of IL-8 and IL-1β, with results showing that IL-8 has no impact on organoid beating rates, which IL-1β resulted in a significant increase in beating rate by approximately 60%.

A disadvantage to this research is that it is not yet at the stage where it can be easily deployed in high-throughput screening. In addition, the researchers did not go more into depth into pharmacokinetics. Additionally, three organoids are only a small representation of the many tissues and organs of the human body. However, this research provides knowledge of inter-organ interactions.

#### 3.1.3. Bead-Based Sensors

Bead-based sensors, although less common, show promise for viable integrated organ-on-chip (OOC) sensors. The next three studies outlined all are focused on bead-based sensors, and although they may also fit into later sections of this review, it is important to join the works together as it will yield a better conclusion as to the methods used in OOC technology.

Non-invasive monitoring is important as it allows for undisturbed observation of the subject at hand. Invasive monitoring, especially with oxygen sensing, may disrupt the biological processes being studied, and consequently alter the trajectory of a particular experiment [117,118]. This next study focuses on the non-invasive nature of a 39b-based sensor created by Zirath et al.

This bead-based oxygen sensor was created from amine-functionalized polystyrene beads that underwent washing, treatment, and calibration before being used [119]. Monolayer cultures were created and consisted of two glass substrates (VWR) bonded together with adhesive film. The chamber height was 460 µm and the chip contained 8 culture chambers of 0.22 cm^2^. Holes for integration were drilled into the upper glass substrate before the sensor particles were introduced. Cultures of A549 lung carcinoma epithelial-like cells, primary human adipose-derived stem cells (ASC), and HUVEC cells were maintained in a 3 °C, 5% CO_2_, and humidified atmosphere. A FireStringO2 optical oxygen meter was used for oxygen monitoring and the integrated sensors were calibrated with a CO_2_/O_2_ oxygen controller. Oxygen monitoring was taken from right after cell solution injection to up to 7 days. A549 cells were seeded at 1 × 10^2^ cells/cm^2^ (2.2 × 10^3^ cells/chamber), 2.5 × 10^4^ cells/cm^2^ (5.5 × 10^3^ cells/chamber), and 1.0 × 10^5^ cells/cm^2^ (22 × 10^3^) cells/chamber). Adhesion promotion of oxygen consumption was investigated by seeding HUVEC endothelial cells on different surfaces, including 5% collagen I and 1% gelatin [119]. An optimized protocol for on-chip oxygen consumption measurements was followed, which included three consecutive cycles of 10 µL/min for 10 min followed by a 3 min recording phase under slow-stop conditions. 

Different tissues were used to evaluate the oxygen monitoring method. The tissues were established cell types and cancer cell lines. The partial pressure was monitored for epithelial (A549), endothelial (HUVEC), and mesenchymal (NHDF and ASC) cells during cell seeding with the same initial cell seeding density [119]. Oxygen depletion values were obtained and shown to be highest for NHDF fibroblast cells, at a partial pressure of 17 hPa, corresponding to the total oxygen consumption of 183 ± 0.4 hPa oxygen during cell adhesion in 3 h. Oxygen consumption for A549, HUVEC, and ASC cells was lower, at values of 104.2 hPa, 28.4 ± 0.4 hPa, and 59.4 ± 15.4 hPa, respectively. Low cellular respiratory activity was exhibited through oxygen consumption values, and the results were consistent with other literature findings, demonstrating the good performance of the microfluidic oxygen sensing method. Through the testing of HUVEC endothelial cells in environments with different surface promoters (1% gelatin and collagen I), it was concluded that the oxygen detection method of the study is applicable for adhesion and biocompatibility studies. The integrated oxygen sensor array was evaluated as a co-culture model of ASC and HUVEC cells. Through the culturing of the co-culture onto an oxygen-permeable PDMS-based biochip, oxygen pressure for the feeding channel remained fairly constant, while the oxygen levels in the hydrogel slowly decreased over time with an average decrease of 2.5 hPa/h. An average partial oxygen pressure of 10 hPa decrease was observed for the deepest hydrogel regions. However, this reduction was shown to not affect the vascular network formation. It was shown that combining optical in-line oxygen monitoring and impermeable microfluidic biochips allows for oxygen control in microfluidic cultures. With this oxygen control, the cultures underwent normoxic and hypoxic cycles, which lead to the formation of vascular networks with an observable thickness of vascular sprouts and a decreased length. Over a 36-h period, partial oxygen pressure recovered to 200 hPa within the nutrient and oxygen limitations; this is a result of cell death from nutrient and oxygen depletion for over 6 days. The results, thus, allowed for the conclusion that the experimental setup allowed for the accurate detection of cellular oxygen demand and dissolved oxygen levels in 2D and 3D microfluidic cell culture systems.

This measurement technique has the potential to be used for indirect monitoring of cell viability in toxicological screening studies. The sensor can monitor complex 3D microenvironments, thus, paving a way for a microfluidic system to control, mitigate, and accurately predict cell behavior. The integrated bead-based sensor offers non-invasive, real-time, and label-free in situ monitoring of oxygen uptake rates, cell viability, and metabolic activity.

To have an in-line measurement of cell-secreted biomarkers, Riahi et al. developed an automated microfluidic bead-based electrochemical (EC) immunosensor incorporating disposable magnetic microbeads(MBs), which cause biomarker-recognition molecules to be immobilized [120]. Microvalves were further integrated into the sensors to allow for the immunoassay to have programmable operations, such as bead loading and unloading, binding, washing, and electrochemical sensing. This sensor was then applied to the liver-on-a-chip platform that would continue to monitor biomarkers secreted from hepatocytes. During a five-day hepatotoxicity assessment, production data on transferrin (TF) and albumin were collected. In conclusion, this research on immunosensor sensors furthers knowledge in the field regarding microfluidic bioreactors and OOCs due to its ability to detect biomarkers in low volumes and analysis of cellular function in long-term in vitro assessments.

Before integrating the sensor into the OOC platform, the researchers conducted a series of experiments to ensure the accuracy of reaction parameters. Results found that the best process consisted of using 7500 MBs for immobilizing 2.5 μg/mL of detection antibody in combination with 1.0 μg/mL of SA-HRP for the EF immunosensor. Next, the microvalves were integrated into the microfluidic immunosensor chip used to detect TF biomarkers with results compared to a commercial ELISA kit to confirm accuracy. Specifically, the data from HepG2 bioreactor results on days 1, 3, and 5 were used for comparison. Their results showed to be comparable to ELISA and HepG2 cells increased secretion rate of TF as time increased. In other words, the researchers were able to track changes in biomarker concentrations continually using small liquid volumes. It should be noted that data collected from on-chip measurements were consistent with ELISA than off-chip measurements, meaning that this confirms the accuracy of the reliability of detection of the target biomarker by the on-chip immunoassay. Another test was conducted to investigate the ability of the EC sensor and three sample solutions were used from the bioreactor on days 1, 3, and 5 to investigate the performance of repeated measurements and assess potential structural defects over time. Results showed that there was no significant difference between measurements when the EC sensor was repeatedly used for 6 days. These consistent results indicate minimal surface and structural defects of the EC sensor. This sensor was then integrated into a liver-on-a-chip platform and resulted in data collection of changes in cell-secreted TP and ALB when APAP treatment was administered. These data were continuous for a long period of measurement and agrees with ELISA analyses. In conclusion, this study demonstrated the performance of selective and reproducible TF detection of a EC immunoassay, which can be integrated into an organ-on-a-chip platform.

This study provides a potential for a new programmable microfluidic bead-based EC immunosensor to be used for OOC platforms. In addition, their study of an on-chip immunoassay revealed a method of detection for biomarker measurements in biological samples to be reliable and cost-effective. A compromise between high sensitivity and reproducibility of measurements demonstrated an optimal number of loaded MBs for the on-chip EC measurements, which agrees with previous studies. Their research can be used by others to further knowledge in this field.

A final study using a bead-based construction was conducted, only this time it was used to detect growth. A cell culture chamber was custom built and placed on a fluorescent microscope (Nikon Eclipse TI) for maintenance of physiological conditions during sensing (35 °C, 5% CO_2_) [121]. Primary rat hepatocytes were used in cell culture. Both capture (non-fluorescent) and detection (fluorescent) microbeads were used in the device for detection, and both microbeads were prepared similarly and some of both types were functionalized with Abs, which could be stored for up to 2 weeks at 4 degrees C. Soft lithographic methods were used for fabrication of microfluidic devices. A silicon master was prepared with patterning SU-8 2050, and then liquid PDMS was poured over the wafer after being mixed with a curing agent. After curing, a biopsy punch was used to make inlet and outlet ports. 80 μm wide channels were created by two columns of PDMS posts, in which the liquid PEG and PBS were fit into the channels, treated, then stored to remove unpolymerized PEG and make the channel surfaces hydrophilic [121]. Numeric simulations were performed to estimate cell-secreted HGF and TGF-ꞵ1 secretion rates. Calibration curves were created before the experiments took place for each cell type. The device was seeded with hepatocytes and cultured in a tissue incubator (5% CO_2_ at 37 °C) for 7 days, with a media change daily. Sensing occurred on days 1, 4, and 7, with media in the sensing chambers being replaced with 50 μL, capturing bead media (7.0 × 10^5^) and detection beads 1.0 × 10^8^). It was presumed that only the GFs that diffused to the sensing chamber during the sensing sessions were detected, which each lasted 90 min long. After the sensing sessions, the media in the cell culture chambers were replaced.

**Figure 3:**
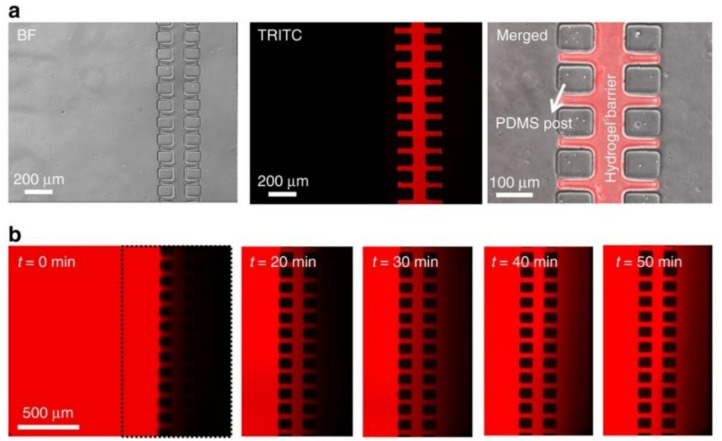
Bead-based sensors to analyze diffusion properties located in the microfluidic chambers for hydrogel barriers, reproduced from open access article Ref. [121] (**a**) The images show the results of bright-field and fluorescence, specifically showing inside the microfluidic device the fluorescently titled 5% poly(ethylene glycol) diacrylate (PEGDA)/20% 20 k poly(ethylene glycol) (PEG) hydrogel barrier. (**b**) Inside the microfluidic device, 5% PEGDA/20% 20 k PEG hydrogel barrier has 75 kDA TRITC-dextran diffused through it. The picture on the left shows a TRITC-dextran (2.5 mg mL^−1^ in phosphate-buffered saline (PBS)) solution flow through the cell culture chamber and hydrogel barrier into the sensing chamber, seen in the picture on the right.

Great effort was put into the optimization of the device, detection, estimation, and reliability. It proved to be challenging to get a proper capture to detection bead ratio (C/D) for optimizing sensing. The C/D ratio was maximized to also maximize the signal to noise ratio of the sensors, resulting in ratios of 1/140 and 32.6, respectively. Microbeads were infused in parallel into the microfluidic sensing chambers to mimic real-life cell-secreted diffusion across the hydrogel barrier. The testing results showed it took 45 min to detect signals, and the signal was stable after 90 min; however, the signal exceeded the noise in the signal to noise ratio by a factor of 3, which set the detection limit for HGF and TGF-ꞵ1 as 6 and 21 pM, respectively (these results were close to those achieved by commercial ELISAs). Local and global concentration gradients were established HGF and TGF-ꞵ1, with results being approximately 7 pM and 0.67 pM, respectively, for the HGF, and 60 pM and 8 pM, for TGF-ꞵ1. When anti-HGF and anti-TGF-ꞵ1 microbeads were introduced and tested in a similar fashion as the HGF and TGF-ꞵ1 microbeads, concentrations of HGF of 7 to 9 pM were revealed, and the concentration of TGF-ꞵ1 detected was about 30 pM. Measurements taken supported the predictions made that the microbead-based sensing of local HGF and TGF-ꞵ1 had higher detection capabilities to the degree of several-folds; however, an eightfold difference was predicted, while only a 4 fold difference was experimentally found. Testing result images can be seen in Figure 3a,b.

There is a need for the long term and local detection of secreted factors within microfluidic channels, and the system provides a potentially easy adaptation to microfluidic systems to accommodate several different cell types and cell-secreted signals. The width of the hydrogel barrier was limited by the fabrication capabilities, and it is an important parameter that governs the permeation of GFs. The strategies developed and tested in this study enabled sensing higher local concentrations of GFs by placing sensing microbeads near the cells, differing from the capabilities of other devices. The device tested and created was able to detect very low secretion levels, which is something other commercial systems are unable to do. The device also proved that the local and global concentrations differ greatly, further shedding light on the importance of precision measurement.

### 3.2. Biomarker Specific Sensors

#### 3.2.1. Oxygen Sensors

Oxygen is a vital element, playing a sustaining role in all sorts of life. Humans functionally operate on oxygen, so the effects of too much or too little oxygen need to be understood as either may give way to a non-homeostatic issue, resulting in compromised health, such as in that seen by hypoxic environment effects [122]. To present the scope and importance of understanding the role of oxygen in the body, it may be beneficial to understand its importance in many processes. Oxygen plays a key role in many chemical reactions taking place in the body, such as pH, blood function, and the Krebs Cycle [123,124,125]. It allows muscles to function, and consequently our bodies to move, and it has been found that more oxygen in the human body allows many bodily processes to occur, which is why a conditioned heart and morbidity are either beneficial or detrimental to one health [126,127].

The studies reviewed in this section focus on works that specifically measured oxygen content and/or the effects of different oxygen concentrations on different biological processes; while no organ or tissue in the human body can hold more than 15% oxygen, many cultures used for drug testing contain oxygen in concentrations of close to 40% [117]. The challenge lies in keeping a low oxygen level, which better represents the body while being able to change media and perform various measurements. This is especially an issue in tumor and cancer research as oxygen concentration has implications for tumor metastasis. Keeping in mind oxygen’s role in life functions, it is easily understood that the detection and characterization of this element through integrated sensors in organ-on-a-chip (OOC)s can lead to technological, medical advances.

To analyze and present a solution to this issue, a device that allowed for precise control and monitoring of oxygen in growth environments was created. Microfluidic cell culture was fabricated in PMMA due to its low oxygen diffusion coefficient and ease of fabrication. Photopolymer resin was 3D printed as the bottom frame support for the 3D culture and also served as the housing for the integrated phosphorescence-based photonic biosensor (iPOB) optical reader. The device consisted of 3 PMMA layers sitting on the 3D printed frame support and topped with a rubber gasket and top lid with gas flow connector ports. NIR Phosphorescent lifetime fluorimetry was used with the iPOB to measure oxygen concentration. A High Concentration Matrigel Basement Membrane Matrix was used to culture the cells; the cultured cells were tumorigenic (BT474) and healthy (MCF-10A) breast epithelial cells [117]. Inside the chamber, gas exchange was simulated by the COMSOL Multiphysics machine. Oxygen concentration was precisely measured and controlled with nitrogen and oxygen exchange in the medium above the tissue scaffold within the device.

Oxygen levels were considered hypoxic if the concentration was less than 2%. Normoxic oxygen concentration was set at 18.6%. The device was tested with no cell, no nitrogen, and no oxygen flow to get a baseline environment reading. The device was then tested with no cells and nitrogen flow, resulting in a significant oxygen concentration drop, thus proving the device worked as it was intended to (the oxygen was purged with nitrogen). When testing the device with the MCF-10 cells, the normoxic and hypoxic oxygen concentrations were 13% and 1.2%, respectively. When testing the device with the BT474 cells, the normoxic and hypoxic oxygen concentrations were 13% and 0.6%, respectively. These results with both test cultures further proved the device’s capabilities to control oxygen concentration and reach hypoxic conditions (<2% oxygen concentration). A higher measure of oxygen concentration was measured on the top of the tissue scaffold when compared to the inside; this remained until both the outside and inside dropped below the hypoxic level. The iPOB has spectral resolution and can identify the effects of tissue scaffold on oxygen transport, as well as monitor the oxygen concentrations. Both cell cultures were kept in hypoxic conditions for 24 h and some hypoxic dependent genes were upregulated.

The device developed (iPOB) was done so to solve the issue of improper environmental concentrations of oxygen, allowing for the complex regulation of oxygen while keeping a relatively stable inner environment. The controlled oxygen environment allows for further understanding of the mechanisms used in cell fate through hypoxic pathways. The iPOB can be used to remotely measure the interstitial oxygen environment when combined with inline analysis techniques, and it can be used to study the complexities of 3D tissue models. Finally, the iPOB can be used to monitor cell culture performance.

To further understand liver pathophysiology, researchers studied three dissolved oxygen (DO) sensors that were inkjet-printed along a channel on a liver-on-a-chip model, named ExoLiver, to observe real-time oxygen [128]. The device consists of two plates, an upper microfluidic channel and a static lower channel with cultivated hepatocyte cells, separated by a porous cell culture membrane. The chip was layered by first applying a SU-8 primer layer, gold electrodes, silver electrodes, and then a SU-8 passivation layer. To find the optimal combination, the researchers tested various amounts of layers of SU-8, silver, and gold, consisting of non-primer (0L), one (1L), two (2L), three (3L), and four layers (4L).

The application of the sensors was tested in three trials: no cells (the control group), rat hepatocyte cells, and human hepatocyte cells. An O-ring silicone surrounded the cell culture area after the three-electrode structures were printed. Located at the top of the plate was a Clark-type oxygen sensor that was used to support the functionality and response of our developed sensors. Every 15 min DO-printed sensor measurements were recorded and correlated with the Clark-type sensor. After a three-hour culture stabilization, all trials added carbonyl-cyanide-4-(trifluoromethoxy)phenylhydrazone (FCCP) to the culture reservoir to increase oxygen consumption from hepatocytes. The medium reservoir was then closed to avoid oxygenation and the system was set under anaerobic conditions. After five hours of exposure to the drug, observations were recorded. The purpose of this study was to determine if there was a presence of an oxygen gradient and to slightly modify/smoothly adapt the membrane to the Liver-On-a-Chip by observing oxygen levels at three different points of the cell culture area (inflow, middle, and outflow).

Testing for an optimal combination of the varying layers was performed first. Results for SU-8 showed that case 1L proved to be difficult to observe because the water droplets were absorbed completely into the membrane. Case 2L showed that the water droplet remained on the sealed membrane surface and the porosity of the membrane was already blocked. Case 3L and 4L showed that the membrane was completely blocked by the polymer. All the cases of silver showed that the primary layer was properly sealed. In all the cases of gold, similar results were observed. Application testing in the control group showed that FCCP had no impact on the behavior of the sensors. In the rat hepatocyte cells trial, data show that between the inflow and outflow, the oxygen gradient was recorded with peak values at 17.5% while the human hepatocyte cells trial measured up a gradient of up to 32.5%. In addition to oxygen gradient, oxygen consumption rate (OCR) was calculated and compared. OCR of the rat cells was 0.23 ± 0.07 nmol/s/10 6 cells and 0.17 ± 0.10 nmol/s/10 6 cells for human cells, which are consistent with other research. When additional doses were added to the rat cells, OCR increased from the first dose to the third dose from 2.33 ± 0.28 nmol/s/106 to 5.95 ± 0.67 nmol/s/10^6^ cells. There was a higher effect that was observed in the human cells after the second dose of 10.62 ± 1.15 nmol/s/10^6^ cells. The experiment is confirmed since the results were consistent with Clark-type sensor data; thus, demonstrating that their DO sensors are functional with rat and human hepatocyte cells cultivated in the lower plate. This research furthers understanding in the organs-on-a-chip field and their results specifically demonstrate reliable real-time sensor monitoring of mitochondrial respiration at different areas of cell culture.

This research showcases an alternative to conventional microelectronic fabrication called inkjet printing (IJP). IJP is a promising, low-cost printing technique that can integrate varying substrates compatible with low-temperature processes. This method reduces the cost of sensors and fabrication time. In addition, this study was able to measure the DO gradient while commercial sensors cannot. The advantage to using these sensors is that through easy configuration the size and shape of DO sensors can be integrated into any membrane, top or lower channel of any OOC system.The following study by Bavli et al. does incorporate an oxygen sensor, and it also incorporates glucose and lactate sensors so that it may effectively bring understanding to liver metabolic function and mitochondrial dysfunction dynamics, a very important area of research as mitochondrial dysfunction may lead to a host of health issues [129,130,131], and is, by definition, an error in the energy creators of cells [132]. Furthermore, reactive oxygen species are known to be a product of aerobic mitochondrial function [133], so the ability to detect oxygen has obvious implications.

In the study, rapid compensation of ATP production due to mitochondrial dysfunction was tested mainly through monitoring glucose, lactate, and oxygen concentration levels [134]. A liver-on-chip device was fabricated and was capable of maintaining 3D HepG2/G3 aggregates for over 28 days in vitro in conditions, mimicking the native liver environment. Two-frequency phase modulation of issue-embedded phosphorescent microprobes was used for real-time measurement of oxygen uptake in liver-on-chip devices. While oxygen uptake was monitored, a computer-controlled microfluidic switchboard that automated washing, calibration, and the amperometric measuring of glucose uptake and lactate production; if mitochondrial dysfunction occurred, a decrease in oxygen and subsequent rise in glucose and lactate would result. The system developed allowed for the detection of very small shifts from oxidative phosphorylation to glycolysis (or glutaminolysis). These shifts were used to test drug concentrations previously believed to be safe via cell mitochondrial stress and dysfunction. Platinum electrodes were used in the amperometric glucose and lactate sensors under a 450 mV polarization and a polymethyl methacrylate bioreactor that permits cell seeding in open configuration was designed to mimic the highly vascularized liver environment in a microwell bioreactor. Off the shelf, medical-grade sensors were integrated into the microfluidic design, which helped permit continual optical and biochemical monitoring.

**Figure 4:**
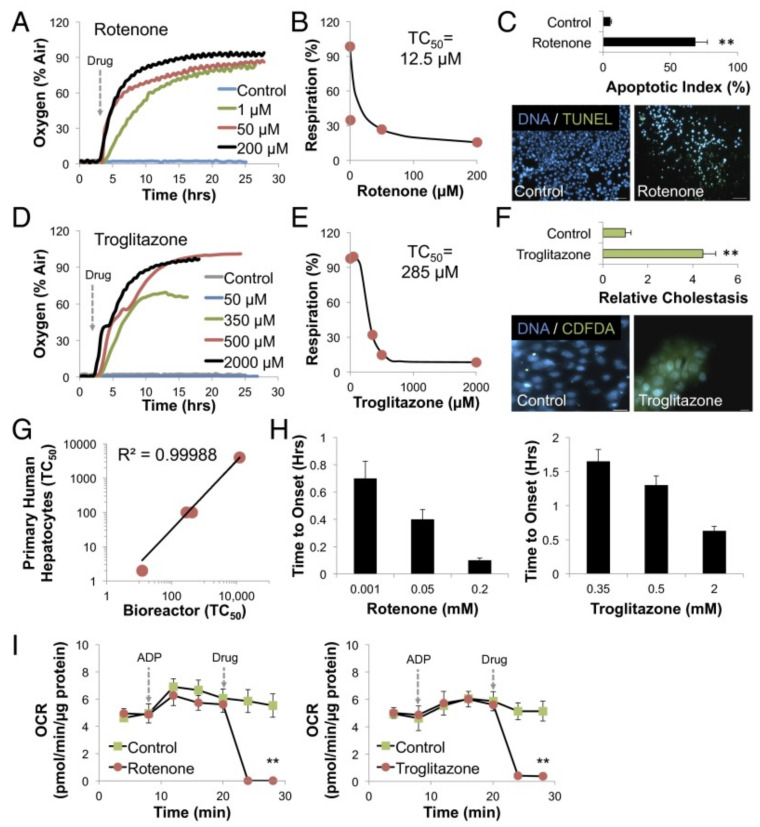
Oxygen sensors for a liver-on-a-chip system, reproduced with permission from Ref. [134]. (**A**) Increasing concentrations of rotenone are administered to HepG2/C3A cells as time progresses in relation to oxygen uptake. (**B**) After 12 h, TC_50_ was found to be 12.5 µM, indicating dose dependence of rotenone. (**C**) Following 24 h of exposure to 200 µM rotenone (*p* < 0.001, *n* = 5), the researchers found that there was a 14-fold increase in apoptosis, found by TUNEL staining, and unlabeled cell death, indicating necrosis. (**D**) Increasing concentrations of troglitazone are administered to HepG2/C3A cells as time progresses in relation to oxygen uptake. (**E**) After 24 h, TC_50_ was found to be 285 µM, indicating dose dependence of r troglitazone. (**F**) Following 24 h of exposure to 200 µM troglitazone (*p* < 0.001, *n* = 5), the researchers found that there was a 4.5-fold increase in the intracellular buildup of bile acids (cholestasis), found by CDFDA staining. (**G**) Curve graph with an *R*^2^ = 0.99 correlation for acetaminophen, amiodarone, troglitazone, and rotenone, displaying TC_50_ values of primary human hepatocytes to their bioreactor. (**H**) The picture on the left shows mitochondrial damage due to dose dependence for rotenone concerning time, while the picture on the right shows the dose dependence of troglitazone. These graphs use the unit of time in minutes instead of hours when describing the exposure of the drugs. (**I**) Shows the relationship of OCR as time progresses. The process included 30 min of isolated mitochondria, then ADP administration, then administration of 50 µM rotenone, shown on the picture on the left. On the right is troglitazone. Through a *t-*test *p* < 0.01, results showed that there was an instant decrease of OCR (*p* < 0.001, *n* = 3), and cytosolic enzyme activation of the drugs were not needed. ** *p* < 0.01 by students's test.

Rotenone, a widely known insecticide and pesticide [135], directly damages mitochondrial complex I, which then induces apoptosis and oxidative stress at low concentrations [134]. During the study, rotenone concentrations (1, 50, 200 μM) were introduced and studied for 24 h, with real-time monitoring of oxygen uptake performed; Oxygen uptake dropped drastically, with only 15% of normal oxygen uptake reported at the 200 μM dose. The oxygen uptake was used to analyze cell viability and metabolic function. Troglitazone, a past antidiabetic and anti-inflammatory drug [136], was perfused at differing concentrations (50–2000 μM) for 24 h, with oxygen concentration measurements taken in a similar fashion for similar purposes as the rotenone. The results were similar to that of rotenone, with a rapid decline of oxygen uptake upon drug delivery, indicating that both can directly inhibit mitochondrial respiration. Quantification for Troglitazone and Rotenone’s effects on mitochondrial dysfunction were further seen by measuring the production of glucose and lactate uptake and production, respectively. There was a very clear drop in the percentage of uptake and production, indicating a shift toward glycolysis, where the glucose is broken down in anaerobic respiration to produce ATP and lactate. However, after six hours of sensing, the lactate over glucose ratio increased (2.6 to 6.1), which suggests that the cells shifted toward glutaminolysis while tending toward apoptosis. Measurements taken also showed glucose was shifted to ATP production. The results show rotenone and troglitazone both can directly inhibit mitochondrial respiration in minutes if there is no enzymatic activity. Images of the results of this study can be seen in Figure 4.

The system fabricated has clear advantages over past OOC devices. It has successfully integrated medical-grade off the shelf sensors in an off-chip unit which drastically reduces costs and allows for the experiment to be continued even if the device needs to be replaced because the sensing device is outside the OOC. The off-chip system also allowed for sensor calibration automation and for the creation of sharp chemical gradients that ensure measurement stability; this is a clear advantage that contrasts previous on-chip glucose and lactate sensors. Another advantage of the device is that it uses optical oxygen measurements, eliminating the oxygen consumption measurement that occurs in techniques like the Clark-type electrode sensing [134]. The sensor integration allowed for the acquisition of data on different metabolic responses of both rotenone and troglitazone leading to mitochondrial damage.

**Figure 5:**
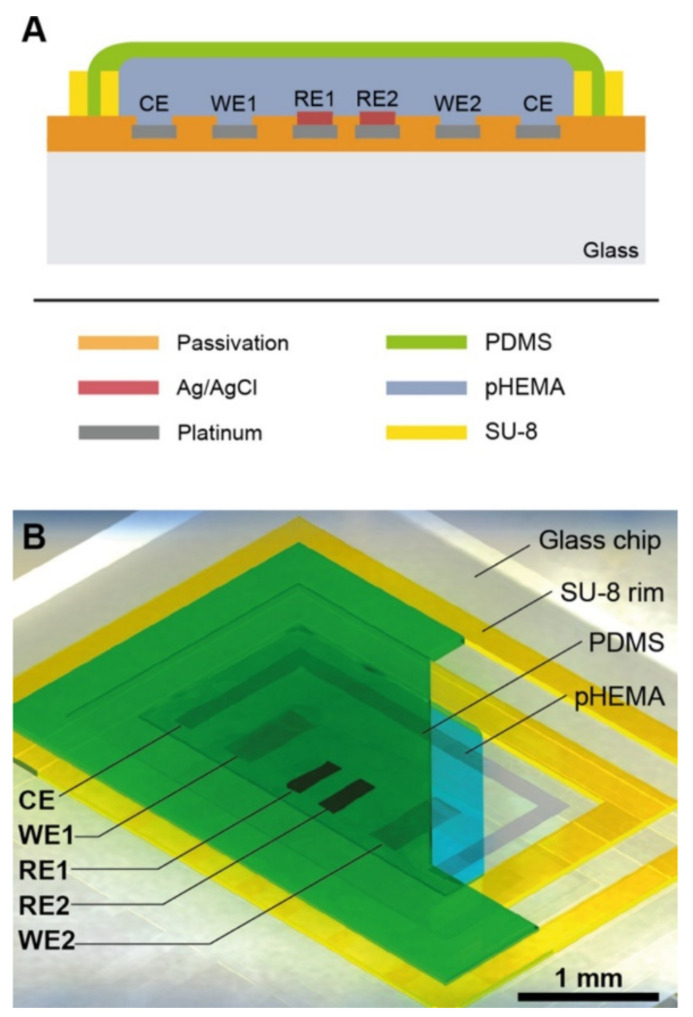
Clark-type microsensor, reproduced with permission from Ref. [137]. (**A**) A graphic showing the sensor (not to scale) including a pHEMA-hydrogel with PBS electrolyte that is surrounded by a gas-permeable PDMS membrane. Additionally, a counter electrode surrounds two Ag/AgCL reference electrodes (RE1, RE2) and two working electrodes (WE1, WE2). The purpose of the SU-8 ring is to define the hydrogel area and create a union of the PDMS membrane. (**B**) Graphic showing the Clark-type microsensor.

This next study took an approach using chronoamperometric protocols that resulted in stable sensor readings for more than a week in air-saturated oxygen concentration for creating stable environments. Thin-film technology was used in the fabrication of glass chips with electrodes, which Clark-type microsensors were integrated with [137]. The electrode layer was a poly 2-hydroxyethyl methacrylate (pHEMA) hydrogel with a phosphate buffer saline (PBS). An electrolyte chamber was created with a permanent epoxy-based photoresist SU8 3025, and then the entire sensor was covered with a hydrogel layer (PDMS) Sylgard 184. The individual chips each had two reference electrodes (WE) (Ag/AgCl), one counter electrode (CE), and two working electrodes, which can be seen in Figure 5A,B. The sensor chips were settled into a 25 cm^2^ tissue culture in a flask. Impedance was measured between the WE and CE with a FRA2 module of the PGSTAT128N system to investigate the dehydration/hydration behavior of the internal electrode. Long term stable sensor operation was achieved by a three-step chronoamperometric protocol. Potentials of the CE were measured and evaluated to understand the corresponding processes of the CE during the Clark-type oxygen sensor operation. The sensor chips were operated for 24 h before the CE potential was measured, and oxygen measurements were taken in air-saturated water at room temperature; a nitrogen flush was optional for comparison with oxygen-free conditions. The sensor was calibrated in BPS at 37 °C. The cell culture was created with T-47D human breast cancer cells; the cells were seeded in a flask with the integrated sensor chip.

The dried-out hydrogel electrolyte allowed for long term dry storage after fabrication. The sensors were reactivated via immersion in an aqueous analyte solution. After hydration, the membrane was ready for operation in aqueous analyte solutions. Measurements of the impedance between the WE and CE at 1 kHz showed conductivity (shown in Figure 2A). The sensors show high impedance values (13 Ω) for the first 18 h, while they are kept in ambient gas-atmosphere or dry conditions. In wet conditions, where the Clark-cells were immersed into DI water, resulted in rapid hydration of the BPS-based electrolyte in pHEMA. After this quick hydration (less and 60 s), slower saturation proceeded and the electrolyte could then be brought into dry conditions and recover the impedance signals. The conclusion of these measurements shows that the sensor can only record in dry conditions for less than one hour. Amperometric measurements at constant potential did not result in stable signals in the oxygen reduction range, resulting in the use of chronoamperometric protocols. In step one of the protocol, a continuous decrease of current was observed (0.8 V to 10 s). Step two involved removing the previously formed PtO (−0.4 V until 20 s). Step three included the establishment of stable oxygen diffusions conditions and the subsequent stabilization of oxygen concentration-dependent current, caused by oxygen reduction. The current peaks for gas formation set in at higher potential, which resulted in a wider water window; this is assumed to be the result of local pH deviation. Minor contamination was also detected in the platinum surface because of the hydrogel membrane presence. Air-saturated analyte potential measurements were recorded to be between −0.55 V and −0.65 V during the first potential step (0.8 V). CE potential measurements in the oxygen-free analyte settled near −1.2 V. Since the pt-H absorption at the CE was insufficient in causing PtO formation at the WE, and state of molecular hydrogen evolution was reached. CE potentials started high and then settled lower (around 0.4 V) in the oxygen-free analyte. Molecular oxygen formation at the CE was indicated by a CE potential of 1.1 V in the presence of oxygen in the analyte. Potential stabilized at the CE in the presence of oxygen in the analyte, indicating continuous production of oxygen. Data from the second increasing ramp in the calibration profile were used to generate a calibration plot, with a sensitivity of −0.121 µAcm^−1^µM^−1^; Each sensor must be calibrated independently, and the sensitivity limit of each was calculated to be 0.2 µM. The setup allowed for stable sensor readings for more than 1 week and the sensitivity of the sensor decreased slightly after 5 days, following an initial increase. Results of this experimental setup show continual oxygen measurements with a 3-electrode setup for more than 1 week, and the specific oxygen monitoring in T-47D breast cancer cells show the microsensors application capabilities.

The researchers in this study thoroughly vetted the current research on Clark-type microsensors’ signal stability, and no evidence was found to support an approach that allowed for a steady signal for more than 2.5 days, while the novel approach is taken, as outlined above, allows for stable measurement for up to 7 days. Although the dry storage capabilities after fabrication are convenient, the sensor operation in a gas environment over longer times is not feasible, even in ambient humidity. Although the microsensor has its drawbacks, it can be easily integrated into other organ-on-chip systems, and it expands the oxygen monitoring time frame with this specific technique.

#### 3.2.2. Metabolite Sensors

Metabolites are simply substances or molecules that are used in metabolism, and they usually are products of a metabolic process. For example, some primary metabolites are amino acids, alcohol, enzymes, and organic acids [138]. They are important biomarkers for processes that occur within the body and have led to great insight into these processes. Past research has shown that metabolites are useful indicators and connections in a host of applications, such as in cancer research [139], immune surveillance [140], and drug safety testing [141], just to name a few. Organ-on-chip (OOC) devices with integrated sensors for various metabolites are reviewed in this next section, and it may be seen that integrated metabolite sensors are another viable way to further the application and effectiveness of these devices.

Researchers have found that many animal models used to predict the toxic side effects of drugs in humans to be inaccurate, so Bhise et al. developed a liver-on-a-chip to better predict the effects [142]. To create HepG2/ C3A hepatic spheroid-laden hydrogel constructs, the researchers fabricated a bioreactor with a direct-write printer, which was incubated for four weeks and encountered continuous perfusion. Once this was complete, cellular functionality was assessed by observing the cellular response to exposure to acute acetaminophen (APAP), which was then compared to in vivo conditions. This experiment lasted for 30 days and data were recorded at the fluidic outlet port using human ELISA kits on days 1, 7, 15, 21, and 30. Data collection took 24 h and average biomarker concentrations in the bulb media were calculated. In addition, calculations of the four biomarkers secretion rates were also found. A series of tests were performed such as the use of an inverted laser-scanning confocal microscope to monitor real-time growth of the spheroids, data collection of cellular metabolic activity, the response to different concentrations of acetaminophen (APAP, Sigma-Aldrich) being administered to encapsulated GelMA spheroids, and testing of the dynamic bioreactor.

There was an increase in cell number per bioreactor observed over the 30-day experimental period from 4 ± 0.5 × 105 cells to 4 ± 0.2 × 10^6^ cells. However, minimum CO2 at any point within the chamber decreased from 91% of the inlet value to 38%. Using Graph Pad Prism software, statistical analysis of the data collected was performed. The results showed that there was a significantly higher albumin production in spheroids, 101 ± 5.2, compared to cell monolayers, 0.5 ± 0.1, these data correlate with other articles, which demonstrated that homotypic cell–cell interactions are potentially improved by culture cell spheroids. In addition, biomarker production was sustained for the entire experimental time due to the bioreactor culture environment. There was an increase in albumin production from 368 ± 62 ng h^−1^ to 400 ± 19 ng h^−1^, transferrin production increased from 181 ± 48 ng h^−1^ to 471 ± 7 ng h^−1^, alpha-1 antitrypsin (A1AT) increased from 185 ± 33 ng h^−1^ to 445 ± 52 ng h^−1^, and ceruloplasmin increased from 35 ± 6 ng h^−1^ to 165 ± 8 ng h^−1^. Similar to other studies, researchers found there to be varying results regarding the metabolic activity and function of cells over time; however, these variations were not statistically significant. Other observations made were an increase in cell number from 4 ± 0.5 × 105 cells to 4 ± 0.2 × 10^6^ cells and a decrease in albumin secreted by the cells in the bioreactor was 20 ± 3 to 2.3 ± 0.1. Although there was a decrease in albumin secretion, it was within a range by a literary article about albumin production per cell by HepG2 cells/ spheroids. Similarly, the amount of A1AT secreted per cell, transferrin, and the amount of ceruloplasmin decreased from day one to day 30. The researchers were able to conclude that their bioreactor responded to the administration of a drug due to the increase in bioreaction condition without APAP treatment and a significant decrease in cellular response when treated with APAP.

As mentioned before, hepatotoxicity is often a result of APAP exposure [31,143]. The model of this study by Bhise et al. was unique in the fact that there was direct access to the hepatic construct anytime due to the ability that the bioreactor could be unscrewed and resealed. Although their spheroids were viable and active during the 30-day experiment period, the results showed a decreasing trend in cellular response, which was suspected to be resultant of declining oxygen availability. They suggested that to solve this problem by increasing the perfusion rate or reducing the size of the spheroids initially.

**Figure 6:**
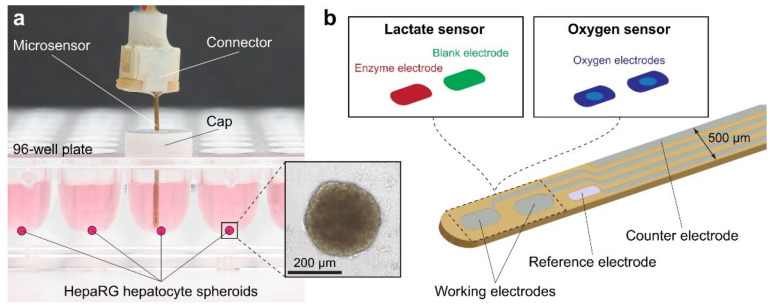
Lactate and oxygen sensors are used to better understand the impact of drugs, reproduced with permission from Ref. [144]. (**a**) The image shows a 96-well cell culture plate holding the microsensor device. (**b**) The graphic shows a magnified version of the tip of the microsensor as well as the electrode layout. Different electrodes are also seen in the graphic such as working and counter. To eliminate unspecific background, the set up uses working electrodes with stationary lactate oxidase and a blank electrode. The oxygen sensor uses the same setup and two working electrodes, which include a limiting diffusion membrane.

This next work, done by Weltin et al., also used metabolite sensors to better understand the effects of drugs, but this time with lactate-specific sensors [144]. To begin the device fabrication, thin-film and laminate technology, along with lithography, was used to pattern different materials and openings, such as those for the sensor platform and electrode and contact openings, respectively. Electropolymerization was used to include a perm-selective membrane, holding back interfering substances at the working electrodes. The processes for the platform were performed on the water level, allowing for cost-efficient and the parallel development of the disposable microsensor device. The length of the devices, after being cut by a CNC, were 16 mm long, 161 μm thick, and around 500 μm wide. The electrode area for the lactate and blank electrodes was 0.095 mm^2^, and the oxygen sensor has a 0.032 mm^2^ electrode area. Hydrogel membranes were used to modify the microelectrodes, allowing for bio- and chemical-sensor integration. Lactate sensors used lactate oxidase entrapped in a poly(hydroxyethyl methacrylate) (pHEMA) hydrogel [144]. A 3-electrode-setup was used to perform all electrochemical measurements. A potential of 450 mV vs. Ag/AgCl with an acquisition rate of 1 Hz was used for amperometric lactate measurements for both working and blank electrodes (a blank electrode was used for calibration purposes). A 3-step protocol was used for chrono-amperometric oxygen measurements using a CompactStat potentiostat at 5 Hz device, with the oxygen sensor switched on every 10 min to avoid oxygen consumption by the sensor itself. HepaRG cells (HPR116) were seeded at 2000 cells/well in a 96 well cell plate and were treated and incubated to form the spheroid culture. The setup used in this experiment can be viewed below in Figure 6a,b. Drug exposure was performed in a serum-free medium with penicillin, streptomycin, human hepatocyte growth factor, and epidermal growth factor.

The sensors showed a sensitivity up to 134 nA m^−2^mM^−1^, which can resolve concentration ranges in the low micromolar range. The detection limit was determined to be between 5 μm and 30 μm. The oxygen sensors were calibrated through adjustments made with different dissolved oxygen concentrations with a gas mixing station. A defined zero point and long-term stable measurements were possible through the chronoamperometric protocol and the diffusion-limited operation mode. It was further proven that interferences like glucose and ascorbic acid have a very minor influence on oxygen measurements due to their slow reaction kinetics and membrane diffusion compared to that of oxygen. Initial lactate concentrations were measured at 48 μM ± 8 μM (*n* = 7). A sensor was placed in the wells while they were sealed and covered for continuous measurement; the results show that lactate production was largely linear over the 70-h test period. The total lactate concentration of 404 μM ± 48 μM (*n* = 7) was measured when the experiment came to an end, with the average lactate production of each spheroid being 356 μM ± 45 μM (m = 7) over the 70 h. These results show the continuous and precise measurement ability of the sensor of a spheroids lactate production over three days. The data exposed an average lactate production of 5.1 μM^−1^ ± 0.7 μM^−1^(*n* = 7). Oxygen measurements became stable after 12 h, with a reduction from the initial measured oxygen concentration to steady-state concentration of −9.4% ± 2.0% (*n* = 4). No hypoxic conditions were detected in any of the wells, thus appropriately bringing the conclusion that lactate production is not caused by hypoxic conditions outside the spheroid. The cells were separately exposed to Antimycin A and Bosentan, a cellular lactate production agent a dual endothelin receptor antagonist, respectively, to test the device’s ability to detect different levels of lactate. The device worked as it measured concentrations appropriate with what was to be expected by the effects of each substance.

An electrochemical microsensor system was integrated into a 3D cell culture, allowing for bio- and chemical-sensors to give access to cellular metabolic parameters in near real-time. The system offered precision and stable measurements of a single spheroid, low concentrations for several days. The single-spheroid long term online measurement in a standard microplate was the first of its kind. The methods and results prove to be promising for continuous monitoring of cells or microtissues in many research applications, such as drug-screening, toxicology, personalized cancer therapy, and further organ-on-a-chip (OOC) engineering. The experimental setup allows for the possibility of mass-fabrication with relatively low costs. The setup also has the potential to be automated and adapted for multiple good measurements in an efficient time manner as stable measurement values can be obtained within a minute of the probe being in the medium. Unstirred vessels sometimes can prove challenging to measure in comparison to a bioreactor because the unstirred wells sometimes develop a diffusion gradient near the electrode. The results in the study show that stable linear signals are obtainable by diffusion through the membrane and potentially adding thermal convection inside the microwell. The online, direct access to 3D microtissue metabolism and culture conditions show advancement in vitro models for toxicology, further facilitating the replacement of animal testing. Furthermore, the systems will allow human in vitro measurement and control models of the metabolic microenvironment, potentially making a tremendous impact in cancer therapy, personalized medicine, and tissue engineering.

Lactate measurements were also taken in the work conducted by Simmons et al. To begin, the group created the device, starting with a scaffold produced from poly(L-lactic acid) [145]. The scaffolds were cut and analyzed, with each fiber being around 24.5 μm in diameter and with an overall average porosity of 88%. MSCs were extracted from rats and isolated from marrow through culturing. The scaffold was prepared for seeding through pre-wetting and then exposed to α-MEM for one hour in a flow perfusion bioreactor. α-MEM was continually perfused through the scaffold after the cells were seeded for the remainder of the culture period (7, 14, or 21 days). The bioreactor media was changed every other day. Fiber-optic probes were used for the oxygen measurements, with specific techniques, equations, and control systems integration for converting the optical measurements into oxygen measurements. A colorimetric glucose assay kit was used to perform a daily glucose assay. Lactate assays were performed every other day on samples of α-MEM using a lactate assay kit. A fluorescent PicoGreen dsDNA array was used for the determination of cellularity of constructs sacrificed at different times; these tests were destructive. The cellularity found was confirmed with DAPI and phallacidin staining and subsequent analysis. Measurements were taken in triplicate and statistical analysis was performed to create a linear regression.

The metabolites of oxygen, glucose, and lactate were studies to quantify cell growth within the bioreactor. Destructive analysis for cellularity determination was performed at various times throughout the up to 21-day test periods. The cell seeding was approximately 29% successful initially, and the cellularity was nearly doubled after 7 days of culture (from 7.5 × 10^5^ to 1.3 × 10^6^). The cells grew slightly for the next 7 days and then decreased by day 21. Oxygen uptake was measured at the inlet and outlet of the bioreactor each day, and the inlet concentration remained relatively constant at 17.45 *±* 1.76%. However, the old media and new media differed significantly in oxygen uptake through the course of the incubation, with the cause likely being the stress of the three-hour culture period in the absence of FBS. Glucose consumption increased after the first week in culture and stayed high for the remainder of the culture period. Cell-specific glucose consumption was calculated and found to be 15 ± 6 pmol/cell/day, which is higher than that found in other studies. However, the difference may be explainable by the potential mitigation within a direct perfusion system, as it may lead to higher values (like those found in the current study). Lactate measurements were taken daily; short periods for lactate accumulation and stress-induced on cells by removing them from the FBS before measuring may have led to more error than reported. Nonetheless, the lactate production went up quickly and then leveled off over the incubation period. Correlation studies between oxygen consumption were conducted to determine scaffold cellularity, and the results were met with linear regression and a high R^2^ value, but low data points only allow for the conclusion that the method for determining cellularity of a scaffold mid-culture is feasible. A correlation study between glucose consumption and cellularity and lactate production and cellularity yielded similar results.

The correlation between the data found and the measurements taken support the idea of metabolic data being used to determine the scaffold cellularity in real-time and without destructive analysis. Data points collected and analyzed support the method but are limited to serve as a proof of concept only. The main disadvantages of the study are the lack of data points and destructive analysis. However, the methods used could easily be adapted to different systems and cell types, potentially allowing for integrating the techniques used to more advanced measurements and devices to obtain true real-time metabolic monitoring.

**Figure 7:**
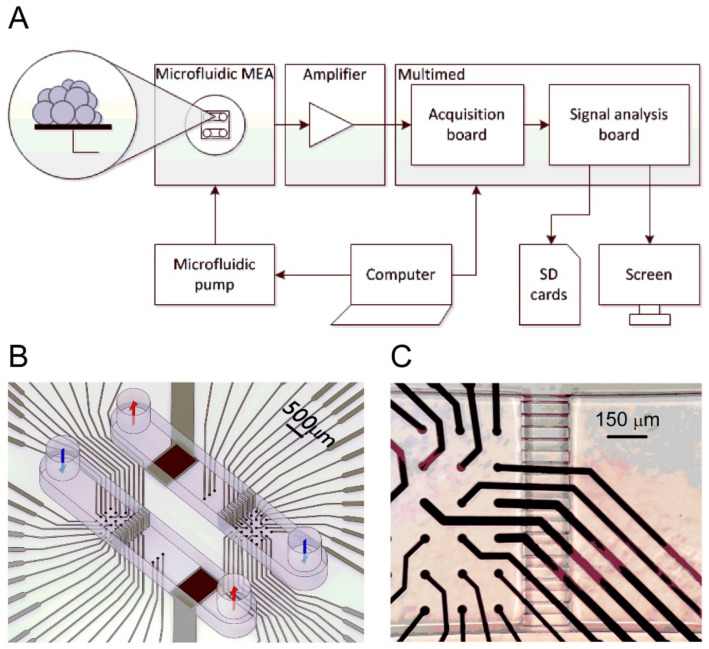
Reproduced with permission from Ref. [146] (**A**) Using a microfluidic pump, perfusion of islet seeds on the microfluidic MEAs occurs. Data of electrical signals are collected, magnified, processed, and analyzed by Multimed acquisition and analysis board. Data that are raw and processed are stored on separate secure digital (SD) cards. (**B**) Microfluidic MEA setup, consisting of two identical chambers in SU-8. Within the chambers, they are further divided into two parts that are separated by a barrier. The barrier is composed of small channels with dimensions of 50 µM. One side has 26 platinum black electrodes with a diameter of 30 µm and a distance of 150  µm between each. The other has one reference electrode and three control electrodes. (**C**) Closeup image of the microfluidic MEA chamber located on the left. In the middle of the image, there is a recording chamber. On the right, there is the reference chamber, outlet, that is separated by the barrier.

Integrating sensors with true real-time monitoring and without lag in real-time measurements lead Perrier et al. to create a device to accomplish the task with analysis of insulin [146]. Experiments were conducted on either standard microelectrode arrays (MEA)s or on microfluidic MEAs (m-MEA). The setup can be seen in Figure 7 below. Adult male C57BL/6 mice were used; after being sacrificed by cervical dislocation, Pancreatic islets were handpicked and obtained by enzymatic digestion. The islets were treated after being gathered. The m-MEA chambers were perfused at 5 µL/min with a microfluidic pump with additional rate control platforms. Within the MEAs, measurements were taken at 37 °C in a buffer with the following, given in mM: 135 NaCl, 4.8 KCl, 1.2 MgCl_2_, 0.2 CaCl_2_, 10 HEPES, and glucose. At each electrode, Signals of the mean frequencies of slow potentials and the amplitude of the envelope of the filtered signal (slow potential amplitude), were recorded on 2 s time intervals. Several additional steps were taken to allow the measurable input to the machines being used to gather the information and read the input. The slow potentials were filtered with infinite impulse response filters. Then, the slow potentials are detected by making local maxima and minima. The results gathered were statistically analyzed with unpaired t-tests with variance. One way ANOVA with Bonferroni post hoc correction was used when different conditions of a single group were compared, while Two-way ANOVA with Bonferroni post hoc correction was used for more than two groups.

To take measurements on pancreatic islets, a two or three-day culture and contact and adhesion between the electrodes and cells are required in classical MEAs. A microfluidic setup was set up to resolve these issues (see Figure 7). The raw data recorded with the setup were displayed on a screen in real-time and stored on a secure digital (SD) card. Each channel within the system consists of two chambers; there is a chamber for culturing and for recording. Extracellular recording of islets gathered action potentials, two electrical signals, and slow potentials, which is representative of a single islet ꞵ-cell activity and reflects their coupling. The focus of the study was on the slow potentials as they are physiologically meaningful in intra-organ coupling. The device was tested for its capacity to discriminate between low and higher glucose levels (G3 vs. G11 and G15). These values were picked for their physiological relevance; 3 mM glucose (G3) is below normal fasting range for both mice and humans, and 11 or 15 mM glucose (G11, G15) is in the high range of postprandial blood glucose levels. The device showed nearly no response, and then vigorous response when low and high glucose levels were introduced, respectively. This demonstrates recordings may be obtained rapidly; sufficient contact between islets and electrodes was established in a short period, contrasting with the several day cultures necessary for classical MEAs. Blind testing was conducted to analyze the capacity of real-time analysis. The experimenter only knew the concentration of G3, but not the three solutions in the experiment (S1, S2, S3). The results of the amplitude measurements show clear, different results through the statistically relevant electrical activity. Despite the narrow range of glucose levels present in the samples (7, 9, and 5 mM), the device was able to correctly rank the concentration of each. The on-line and real-time measurements are significant, as other systems do not allow this. This kind of recording was made possible by the computational algorithms embedded in the reconfigurable Field-Programmable Gate Arrays.

The methods used in this study prove superior in gathering glucose level results, visual inspection, and real-time on-line analysis. Furthermore, the approach provides on-line real-time analysis, eliminating the analysis of off-line large information, and it can be incorporated into a closed-loop. More generally, the approach of this study promotes the capabilities of microelectronics in biosignal analysis and treatment. No other system allows for implant-scaled miniaturization and maintains the real-time latencies. Some hardware devices exist that permit real-time analysis, but in comparison, they are not versatile as they are limited in their degree of configurability. Another advantage of the device in this study is that it is compatible with commercial MEAs. This study falls short in long term monitoring analysis of glucose levels. The islet measurements were taken after the first two hours of the postprandial period, and further development would need to be done to do a real-time, long-term analysis of glucose levels.

The final metabolite sensor shows improvement and promise for understanding the metabolism of tissue. Murine C2C12 skeletal myoblast cells were used for the cell culture; they were expanded in growth medium and cultured at 37 °C and 5% CO_2_ [147]. Prepolymer precursors were synthesized and prepared, with final concentrations of GelMA, CMCMA, and LAP established at 5%, 1%, and 0.1% (*w*/*v*), respectively. The swelling analysis was performed by placing the prepolymer solution in a 48-well plate, exposing it to UV light, rinsing with PBS, and then measuring their weight. The weight was then determined after days 1, 3, and 7, and the mass increase was calculated and the swelling ratio of each hydrogel was determined mathematically. Each hydrogel was prepared into three samples and stress and strain tests were conducted in triplicate. Degradation analysis was performed on each hydrogel after the swelling analysis was complete. The hydrogels were placed in a 6-well plate after being removed from the 48-well plate, and they were left to swell for 3 days. An amount of 3 mL of collagenase type II was added to the hydrogels as they were incubated (37 °C, 100 rpm shaking conditions). The percent of hydrogel remaining was mathematically calculated. The cells were introduced into the hydrogels and incubated within the microdevice with a growth medium for 6 days. After the 6 days, the medium of the culture was switched to differentiation ion medium (DM) to promote myotube formation. Bright-field microscopy images after 6 days of encapsulation were used to obtain aspect ratio, circularity, and other cell description data of the C2C12 cells in GelMA-CMCMA hydrogel. Confocal microscopy images were also taken and cell viability percentages of living to total cells were calculated. The filamentous actin (F-actin) was stained while they were incubated in rhodamine-phalloidin 480. Concerning the device fabrication, interdigitated electrodes were patterned by photolithographic procedures into ITO-glass substrates. The substrates and IDEs were then treated. The PDMS chip was created by mixing a prepolymer with a curing agent (10:1 ratio), degassing the mixture in a chamber for 1 h, and then pouring it on a master mold. After curing, the PDMS chip was bonded to the ITO_IDA electrode substrate through oxygen plasma activation at 10.5 W for 30 s. The SPGE sensor was functionalized by using a self-assembled monolayer(SAM) to prepare it to capture the IL-6 and TNF-α. After the SPGE electrodes were completely prepared, they were connected to a potentiostat and amperometric signal for recording. Electrical simulation was performed via a wave generator connected with metallic connections to a microdevice. Accuracy evaluation was performed. A µSTAT 200 potentiostat was used to gather amperometric measurements. The measurements were acquired under static conditions with the static PMMA cell. Finally, statistical analysis was performed to evaluate the measurements.

The hydrogels were treated with non-biodegradable methacrylate carboxymethyl cellulose (CMCMA) to obtain a hydrogel composite that would not be degraded by cell metabolism. The GelMA and GelMA-CMCMA were exposed to UV light (365 nm) for 18, 24, 30, 60, and 120 s, with energy dodge directly measured as 0.30, 0.37, 0.48, 0.96, and 2.1 J for the exposure times, respectively. It was found that longer exposure times increased the stiffness of both hydrogels (0.2 to 1.6 kPA for GelMA and 0.3 to 3.9 kPa for GelMA-CMCMA). GelMA-CMCMA had a much higher compression modulus compared to GelMA, and the swelling ratio was found to be inversely related to the stiffness. H2O uptake was higher in GelMA-CMCMA compared with GelMA (283 to 58 and 46 to 18, respectively). These results lead to the conclusion to use the GelMA-CMCMA for the tests. UV light exposure for a 24-s duration resulted in the best cell morphology score due to its high aspect ratio (2.149 ± 0.970 and low circularity (0.366 ± 0.188). The cell staining allowed for the assessment of cell differentiation and maturation. Cells were shown to elongate and align throughout the entire 3d muscle microtissue thickness at day 10. The 3D microgrooves were found to restrict cell alignment to between 0 and 15 degrees. The maturation was tested by staining the cell with myosin heavy chain (MHC) and showed that the maturation of microtissue enhanced myotube maturation and cell fusion when compared to the non-patterned cells assessed y MHC staining and fusion index (75 ± 7 vs, 50 ± 3). The on-site multiplexed sensor system allowed for cytokine detection as the enzymatic reaction produced changes in surface electrode current density, which was directly related to the cytokine amount detected by the primary antibody. Enzyme-linked immunosorbent assays (ELISAs) were performed to validate the two commercial antibodies in the study. A limit of detection (LOD) for IL-6 and TNF-α of 0.031 ± 0.010 ng mL^−1^ with a slope of 0.726 ± 0.008 and LOD of 0.060 ± 0.021 ng mL^−1^ with a slope of 0.498 ± 0.012, respectively. After more measurements, calibration curves for IL-6 and TNF-α showed a LOD of ~8 ng mL^−1^ and ~2 ng mL^−1^ with effective concentration 50 of ~78 ng mL^−1^ and ~20 ng mL^−1^, respectively [148]. Four parameter logistic regressions of the data were performed and both assays were shown to have satisfactory accuracy. Cytokine secretion levels were determined by ELISA. Cell cultures were separately incubated with caffeine, dexamethasone, and LPS, where caffeine and dexamethasone showed the highest release of IL-6 in the medium. Similar results were obtained with TNF-α. From these results, LPS was used as the biological agent for inducing cytokine release in the sensing system. Cultured underwent cycles of 1 h simulation followed by 1-h relaxation. The relaxation simulated exercise training, and for the IL-6 and TNF-α, 1 µg mL^−1^ and 10 ng mL^−1^ were detected. TNF-α concentrations increased slowly, but constantly, throughout the experiment, unlike the IL-6 concentrations. Thus, the secretion of cytokines in SM was demonstrated as being regulated by exercise stress. The results suggest SM cells may have a potential role in the myogenic process.

Few examples in published literature incorporate both a microfluidic platform and a biosensing system together to study the metabolism of tissues. The literature that does exist either presents less robust and sensor readings that are highly volatile to medium change, or systems that use methods that reduce the number of measurements acquirable. The transduction system used in this study bypassed both of those obstacles [147]. The new technology developed represents an important step for biomimetic muscle platform development in monitoring skeletal muscle cytokine functions.

## 4. Application of OOC Devices with Integrated Sensors

These final study outlines highlight very specific applications that fall out of the previous, more broad classifications. Furthermore, the studies show promise for specific resolution and understanding of a particular issue, namely muscular dystrophy, and inflammatory arthritis. These studies serve as a conclusive hope in the sensor integrated organ-on-chip (OOC) world, as they effectively realize much of the potential discussed for this technology as a while.

Firstly, Electrochemical sensors were utilized in a study on Duchene Muscular Dystrophy (DMD), a neuromuscular disorder that reduces dystrophin [148]. The following study by Caluori et al. was able to gain insight and side effect understanding of Verapamil, showing, for the first time, a distinct relationship of beating-force for Duchene muscular dystrophy cardiac modes [149].

Cardiac clusters were obtained using human embryonic stem cells (hESC) and induced pluripotent stem cells (iPSC) from a patient with DMD. The cells were tested for the presence of sarcomeres with a striated pattern and labeled with cardiac troponin T and the presence of dystrophin in the cytoplasm and then were seeded on mitotically inactivated mouse embryonic fibroblasts, which served as the feeder cells. Embryoid body formation started cardiac differentiation. After further cultivation, beating cardiac clusters (BCCs) were seen on day 14, and then were transferred from a hypoxic atmosphere to a normoxic atmosphere. The BCCs were kept in a medium with 10 µg ml^−1^ ascorbic acid after day 22. Microelectrode Arrays (MEAs) were prepared before the beating CMs were plated. Through the cleaning and sterilization process prepared the MEAs to be treated with a laminin and fibronectin mixture to promote cell growth after which they were put in an overnight incubator (37 °C, and 5% CO_2_). BCC samples were manually placed on top of the electrode arrays before being incubated overnight. The BCCs were allowed 3 days to stabilize in order to ensure proper adhesion, spreading, and cell-electrode contact. The MEA electrodes were interfaced with an electronic board with 60 channel unity-gain amplifiers. The thermal noise method was used to calibrate the cantilever spring constant before the cantilever was immersed in a liquid and put in contact with the sample to identify the superficial contraction center. After the center was found, drug trials began with 5 min recording and 10 min settling time intervals. Drugs were incrementally added, but never exceeded 5% of the total volume in the MEA chip (2 mL). All the experimental protocols were repeated on three biological replicates of each cell line. A LabVIEW virtual instrument aided in recording the electromechanical data, which was acquired at a 5 kHz sampling frequency. The data were post-processed with MATLAB R2016b, with statistical analysis performed with Prism 5.0. A 1-way ANOVA with Bonferroni post-test was used to test for statistical significance.

The setup allowed for electromechanical recording through simultaneous visualization, coupled analysis, and synchronous recording. The average noise in force and Z height traces were 18.3 nP and 24.5 nm, respectively. A visible EFT trace was produced by about half of the plated samples during the mechanical beating. This number is fairly low yield and is attributed to scarcely conductive coupling between the BBC and MEA planar electrodes. The samples could be kept without visible contamination for up to 4 days if they were filled with an antibiotic-containing medium, which is particularly useful for several day treatments. Forces of 5 nN were applied and did not produce any spontaneous change in the measured parameters. Beating rate, cardiac cycle duration, contraction speed, and a coupled parameter like that of the electromechanical delay (EMD) were able to be reconstructed within the apparatus and recorded. Baseline differences of beating frequencies were not significant, which allowed for the conclusion that the basal conditions would be sufficient to realize differences between healthy and diseased hPSC-derived BCC. Cardiac parameters on extracellular Ca^2+^ concentrations were tested as the Ca^2+^ serves an important role for ECC, especially for hPSC-derived CMs. A 2-way ANOVA test was used to find cell-type dependency, with results of *p* = 0.007 for contraction rate, *p* = 0.0003 for contraction duration, *p* < 0.0001 for time decay, and *p* = 0.026 for EMD. Ca^2+^ concentration effects were found to be significant only in relationship with the beating rate (*p* < 0.0001) [149]. All the cell lines experienced a beating rate decrease proportional to the Ca^2+^ concentration addition, and the decrease was parallel with an increase in exerted force in all the cell lines (120.3 ± 9.4% for the control group, 110 ± 14% for DORSO, and 112 ± 19% for DMD). Control and DORSO showed no dose-dependent relationships with the Ca^2+^ concentration, with various ranges of 109–99% and 142–131%, respectively, contrary to the DMD group, which showed a dose-dependent trend up to 257 ± 71% of the basal delay [149]. Ꞵ-adrenergic stimulation was performed with increasing concentration (182 ± 29% and 186 ± 22%, respectively). The DORSO and control groups experienced only about ⅓ and ⅕ of the increase, respectively. Verapamil served as a class IV antiarrhythmic and was shown to improve skeletal muscle force [150,151] in DMD patients, but negative cardiac side effects also resulted [149,152].

The CBB proved to be robust with line-specific variability in differentiation efficiency through obtaining results that no dose-dependence with Ca^2+^ concentration was observed for the control. The study was able to gain insight and side effect understanding of Verapamil and it was able to show for the first time a distinct relationship for beating force for DMD cardiac modes. While these discoveries are significant, the system lacked in its measurement environment sterility, and only a single MEA electrode and AFM cantilever were used for 3D model probing, leaving the potential for greater variation than what was detected. 

**Figure 8:**
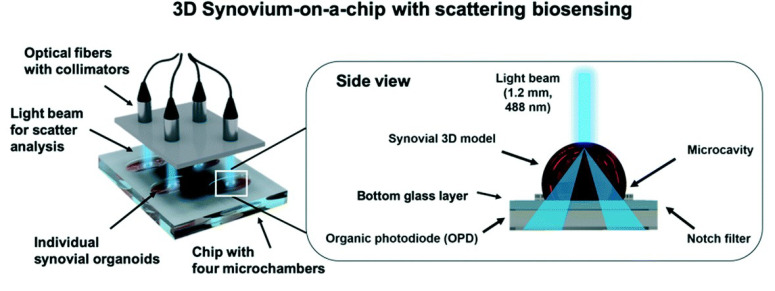
Investigation of tissue-level remodeling in regard to inflammatory arthritis, reproduced from open access article Ref. [153]. Graphic showing setup of the chip with integrated biosensors, consisting of four separate microchambers that hold human synovium organoids. Using organic photodiodes below the chip, light scatter measurements are recorded through the separate synovial organoids.

A final study to be outlined in this review was conducted by Rothbauer et al. that used a light scattering platform for synovial organoid development and analysis [153]. The light scattering station consisted of multiplexed 488 nm sapphire laser, split by beam splitters, collimators, and fiber couplers. The light was scattered at an angle greater than 20 degrees to the incident beam. The light passing through the notch filter was detected by an organic photodiode, creating an electrical signal as potential difference,*n* which can be seen in Figure 8. The potential difference was recorded with LabView GUI. Human synovial tissues from patients with Rheumatoid Arthritis (RA) were used. Human fibroblast-like synoviocytes (FLS) were isolated and cultured for their experiment. After further treatment, the cells were split in a 1:3 ratio, with medium changed every week. Tumor necrosis factor-alpha (TNF-α) was incubated with the arthritic phenotype synoviocytes for biochemical stimulation. The biochip was made from three layers of microscope glass slides bonded by biocompatible pressure-sensitive adhesive tape. Hydrophobic PDMS structures were used to house and hold the three-dimensional synoviocyte organoids. The chips were further treated and then incubated at 37 °C for 35 min for gelation. For analysis of inflammatory stimulation of the synoviocyte cultures, a human IL-6 uncoated ELISA was used. Cell viability was evaluated through live-dead fluorescence staining. Absorbance measurements were performed using a plate reader at 535 nm. Further histological analysis was performed on the data.

Light scattering signals (voltage) revealed a linear voltage increase as a function of particle concentration ranging from 125 µg mL^−1^ to 1 µg mL^−1^ to and size 46 nm to 10 µm. The particle numbers ranged from 1.25 to 5 × 10^5^ total particle count. Experiments with increasing patient-derived primary FLS synoviocytes embedded in the Matrigel hydrogel showed the linear signal rose over the dynamic ODP sensor voltage range of 0 to 10 V; this yielded an approximate 500 cell µL^−1^ detection limit, with a sensitivity of 1.9 mV. The apparatus also allowed for readily adjust detection range and sensitivity to any tissue structure. Light scatter signals gathered over four days of pure Matrigel, 2D synovial fibroblast culture, and hydrogel-based synovial organoids were compared and the comparison showed that in the presence of pure Matrigel, there were stable light scatter signals. Significant signal changes were observed in a hydrogel under 3D conditions over the four-day cultivation period; for the first two days, a linear increase was observed, followed by a plateau at day 3, indicating a lining layer was created and a complex 3D cellular network was created. Scattered light voltages obtained demonstrate that on-chip organoid growth within the PDMS structure is key for reproducible measurements on the synovium-on-chip station. The 3D light-scattering method was determined to be suitable for biological applications as no detectable loss of cell viability. Histology analysis and fluorescent microscopy aided in establishing physiological synovial architecture related to healthy synovium within the organoid. To further support findings of healthy synovial organoid growth, deposition of collagen fibers at day 4 post-seeding was visible through second harmonics generation microscopy. TNF-α smented the culture media during cultivation to recreate the pathology of inflammatory arthritis. In the presence of TNF-α, the light scattering signal showed an increase of 16% and 21% at days 3 and 4, respectively. The increase is likely attributable to synovial organoid structural changes. Cell viability and metabolic activity remained fairly similar in both treated and untreated stimulation. Overall, cytokine increase in TNF-α organoids was observed when compared to the untreated group. In overall tissue-level morphology analysis, a thickened hyperplastic synovial lining layer in the TNF-α stimulated organoids were identified, indicating the presence of an Inflamed phenotype of the synovial tissue. Increased cell migration and changes in cell-to-cell interactions were seen after TNF-α stimulation in the center region of the organoid.

Current testing for human arthritic diseases is conducted in large within animal test subjects. The process poses many issues and complications, with a major one being throughput, as the animals being injected require highly skilled personnel to reduce pain and suffering, as well as accurately insect and induce the arthritic disease to be studied. This makes understanding very difficult and progresses very slow in the field of arthritic diseases, but the ability to create and test synovial organoids in an organ-on-a-chip device could solve the issues and lead to further understanding and medicine improvements. The study showed a diseased phenotype can be distinguished in as little as 2–3 days post-seeding, which is far better than the 13–21-day distinguishing period post-seeding for conventional models.

## 5. Conclusions

Organ-on-chip devices with integrated sensors continue to provide invaluable insight for pharmacological testing and advances in engineered tissue. Technology can advance, and the quality of life improves, as these MPS pave a path for better understanding. Through the many works outlined in the paper, it was seen that there is much work being conducted with these devices, with the main goals of eliminating animal testing, improving the throughput of effective drugs (while consequently quickly eliminating dangerous drugs), and create more biologically relevant engineered tissue.

However, a drawback in many of these studies is that of legitimate application. While the research being conducted is yielding advancement in OOC technology and the integrated sensors, nearly all the works hinted at the potential of the devices, not their application. Many recent efforts analyzed made clear technological improvements, but they still lacked in field efficacy. The next generation of OOCs and their integrated sensors need to move toward application. The technology is being developed rapidly, and the functionality, as was noted in the above text, is often created to be easily accessed and adaptable, which leaves the hope that these technologies will be more applicably utilized. It would seem there is a repeat in the cycle of research that breads some new technology and much potential, leaving the world with a vat of possible applications and only a small bucket of legitimate uses. In this way, the research in this field seems to mimic the path of a newly developing drug. Many trials are made and most fail, with only a handful proving effective and safe. Now that a wealth of integrated sensor technologies exists, they ought to be used to break the cycle they were created to eliminate in the first place.

After the careful review and analysis in specific and at large of the current technology, the next generation of sensor integrated OOC devices will likely yield purposeful and strong steps toward eliminating animal testing and improving biomaterials. Mechanical, electrical resistance, and conjunctive systems that use a combination of sensors were shown to effectively characterize cellular parameters and work well as integrated sensors. Furthermore, it was seen that these sensing methods, with other methods, were used to measure the specific biomarkers of oxygen and various metabolites. Finally, it was seen through specific applications that these sensors not only pave potential for advancement, but they are used for the legitimate analysis of biological processes, and thus lead to the conclusion that the OOC devices are advancing to be more relevant and useful in the medical and engineering worlds. Thus, the hopeful potential does not need arrest, as the work being conducted is showing that organ-on-chip technology is promising for improving lives around the world.

## Data Availability

Data sharing not applicable to this article as no datasets were generated or analyzed during the current study.
